# RLM1, Encoding an R2R3 MYB Transcription Factor, Regulates the Development of Secondary Cell Wall in Rice

**DOI:** 10.3389/fpls.2022.905111

**Published:** 2022-05-31

**Authors:** Zhenhua Chen, Shouzhen Teng, Di Liu, Yuan Chang, Liying Zhang, Xuean Cui, Jinxia Wu, Pengfei Ai, Xuehui Sun, Tiegang Lu, Zhiguo Zhang

**Affiliations:** ^1^Biotechnology Research Institute, Chinese Academy of Agricultural Sciences, Beijing, China; ^2^College of Food Science and Biology, Hebei University of Science and Technology, Shijiazhuang, China

**Keywords:** rice, RLM1, R2R3 MYB transcription factor, secondary cell wall, yield

## Abstract

Leaf morphology is an important component of rice ideal plant type. To date, many regulatory genes influencing leaf morphology in rice have been cloned, and their underlying molecular regulatory mechanism has been preliminarily clarified. However, the fine regulation relationship of leaf morphogenesis and plant type remains largely elusive. In this study, a rolling-leaf mutant, named *rlm1-D*, was obtained and controlled by a pair of dominant nuclear genes. Cytological observations revealed that the *rlm1* was mainly caused by abnormal deposition of secondary cell walls. Molecular evidence showed ectopic expression of a MYB-type transcription factor LOC_Os05g46610 was responsible for the phenotype of *rlm1-D*. A series of experiments, including the transcription factor-centered technology, DNA-binding assay, and electrophoretic mobility shift assay, verified that RLM1 can bind to the promoter of *OsCAD2*, a key gene responsible for lignin biosynthesis in rice. An interacting partner of RLM1, OsMAPK10, was identified. Multiple biochemical assays confirmed that OsMAPK10 interacted with RLM1. OsMAPK10 positively regulated the lignin content in the leaves and stems of rice. Moreover, OsMAPK10 contributes to RLM1 activation of downstream target genes. In particular, *RLM1* is exclusively expressed in the stems at the mature plant stage. The yield of RLM1 knockdown lines increased by over 11% without other adverse agricultural trait penalties, indicating great practical application value. A MAPK-MYB-OsCAD2 genetic regulatory network controlling SCW was proposed, providing a theoretical significance and practical value for shaping the ideal plant type and improving rice yield.

## Introduction

Leaf morphology plays an important role in rice ideal plant type. Leaf morphology improvement focuses on erect leaves and moderately rolled leaves. Moderate rolling can keep rice leaves upright with no drooping, improving the light reception conditions at the base of the canopy at the middle and later stages, increase the light energy use rate, and enhancing root activity to improve lodging resistance (Zhang et al., 2009; Zou et al., 2011). Some rolling-leaf cultivars, such as Liangyoupeiyou and Miyang, have become popular with growers (Wang et al., 2003). Therefore, the study of leaf morphogenesis has become the focus of breeding for high-yield rice.

Leaf morphogenesis is a complex developmental process that is influenced by many factors. In *Arabidopsis*, the genetic regulatory network of leaf morphogenesis is relatively well known. HD-Zip III, YABBY, and KANADI are involved in the establishment and development of leaf polarity (Emery et al., 2003; Ha et al., 2010). By regulating the expression of HD-Zip III and *ARF3/ARF4*, miRNA165/166 and ta-siRNA, respectively, are also involved in the establishment and development of leaf polarity (Hunter et al., 2006; Merelo et al., 2016). To date, more than 10 regulatory genes controlling leaf morphogenesis have been cloned in rice, and their underlying molecular regulatory mechanisms have been preliminarily studied. The genes involved mainly include *LC2* (Zhao et al., 2010), *OsHB1* (Itoh et al., 2008), *ACL1/ACL2* (Li et al., 2010), *Brd1* (Hong et al., 2002), *CLD1/SRL1* (Li et al., 2017); *Yab1* (Dai et al., 2007), *OsCSLD4/NRL1* (Hu et al., 2010), *OsAGO7* (Shi et al., 2007), *RL14* (Fang et al., 2012), *CFL1* (Wu et al., 2011), *SLL1* (Zhang et al., 2009), *ROC5* (Zou et al., 2011), *ROC8* (Sun et al., 2020), and *PSL1* (Zhang et al., 2021). These genes mainly play a role in regulating the number and size of bulliform cells. *RL14* and *PSL1* positively regulate the development of bulliform cells, while *Brd1*, *Yab1*, *RL14, LC2*, *ROC5, ROC8*, *OSHb1*, and *ACL1/ACL2* negatively regulate the development of these cells. In addition to the size and numbers of bulliform cells that cause leaf rolling in rice, the distribution of mesophyll cells, the formation of sclerenchyma cells, and the development of leaf cuticles can affect leaf rolling. *SLL1* encodes a KANADI family MYB transcription factor. Mutation of *SLL1* leads to a disruption of the programmed death process of abaxial mesophyll cells and inhibits the normal formation of abaxial sclerenchyma. *CFL1* encodes an unknown protein with a WW domain and promotes leaf rolling. CFL1 interacts with HDG1 (HD-ZIP IV) proteins and negatively regulates the development of cuticles by affecting the expression of *BDG* and *FDH* genes related to cuticle development. Totally, leaf morphology underlying molecular regulatory mechanism has been preliminarily clarified. However, the fine regulation relationship of leaf morphogenesis and plant type remains largely elusive.

To better understand the molecular mechanism of leaf morphogenesis and plant type, the rolling-leaf mutant *rlm1* was studied in depth. The *rlm1* is controlled by a pair of dominant nuclear genes. Cytological observations revealed that RLM1 directly affects secondary cell wall (SCW) deposition, indirectly regulates the development of bulliform cells, and promotes leaf rolling. *RLM1* was cloned by a map-based method and found to encode an R2R3 MYB transcription factor. The *rlm1-D* mutant phenotype was caused by the ectopic expression of the R2R3 MYB transcription factor. Combining biochemical, cytological, and genetic strategies, we proposed a new molecular regulatory network based on MAPK-MYB-OsCAD2. This study improves our understanding of the relationship between leaf morphogenesis and plant type and provides genetic resources for cultivating ideal plant-type rice materials.

## Materials and Methods

### Plant Materials and Growth Conditions

The *rlm1-D*, WT, overexpression lines RLM1-OE1 and RLM1-OE2, mutant lines *RLM1-m1* and *RLM1-m2*, and plants used for mapping populations were grown in the greenhouse and at a field station of the Chinese Academy of Agriculture Science. All the plants tested at the seedling stage were grown in a growth chamber with 10 h daytime/14 h night-time photoperiod at 28°C, under a light intensity of 150–200 μmol/m^2^/s and under 60–70% relative humidity (RH).

### Mapping of RLM1

A mapping population was generated by the crossing of *rlm1-D* heterozygous plants with the *Dular* cultivar (*Indica*). Ten WT leaf samples were collected for preliminary mapping. Nearly 500 plants were used for fine mapping, and the location of *RLM1* locus between Indel2670 and Indel2672 was narrowed. A T-DNA insertion was found in the promoter region of LOC_Os05g46610.

### Gene Overexpression and CRISPR-Cas9

RLM1-pCAMBIA1302 vectors containing the full RLM1 ORF were constructed to reproduce the phenotype. Two Cas9 targets were selected *via* CRISPR-gene editing (GE),^1^ and RLM1-pP1C.7 was constructed. The full-length OsMAPK10 CDS was cloned into pCAMBIA1302, and two Cas9 targets were selected *via* CRISPR-GE to generate the OsMAPK10-pP1C.7 knockdown plasmids. All the plasmids were then transformed into the *Agrobacterium* AGL1 strain, which were then transformed into *Nipponbare* calli.

### Analysis of Expression Patterns by qRT-PCR and GUS Staining

RNA samples were isolated from WT plants at the seedling and heading stages. The cDNAs were synthesized *via* FastKing gDNA Dispelling RT SuperMix (KR118, TIANGEN, Beijing). qRT-PCR was subsequently performed with SYBR qPCR Mix (ZF501, ZOMANBIO, Beijing) according to the manufacturer’s instructions. The housekeeping gene *OsActin1 (LOC_Os03g0718100)* was used as an internal control to normalize gene expression.

ProRLM1-1391Z vectors were used to determine the expression pattern, and the 2,000 bp sequence upstream was amplified from the RLM1 codon start site. The recombinant pRLM1-1391Z *Agrobacterium* strain was subsequently transformed into *Nipponbare* calli. At the seedling and heading stage, GUS staining was conducted, with 2 mM X-Gluc applied to different tissues.

### Subcellular Localization of RLM1

The full-length CDS of RLM1 was cloned, amplified, and inserted into pAN580. The resulting RLM1-pAN580 constructs were transformed into *Nipponbare* protoplasts, and mCherry was selected as a nuclear marker. The signals were detected using a laser scanning microscope (LSM980, Zesis, Germany).

### SEM Observations

Stem samples from leaves or the 2nd internode were collected from rice at the heading stage. The tissues were cut to 0.5 cm and fixed in 2.5% glutaraldehyde for 12 h. The samples were prepared according to the methods of Wang et al. (2022) and then imaged with a scanning electron microscope (H-7500, Hitachi, Japan).

### Lignin and Cellulose Content Measurements

Samples of leaves and stems from WT, *rlm1-D*, RLM1-OE, *RLM1-m*, *OsMAPK10-m*, and OsMAPK10-OE plants at the heading stage were collected. The lignin and cellulose contents of 2 g samples were quantified by Wuhan Metwell Biotechnology Co., Ltd. (Wuhan, China).

### Paraffin Sectioning and Observations

Leaves from *rlm1-D* and WT plants were collected and fixed in Carnoy’s solution (ethanol/glacial acetic acid = 3/1) overnight. The samples were then treated according to the methods of Sun et al. (2020) and observed as previously described (Cui et al., 2019).

### Phylogenetic Analysis

A BLAST search of *Arabidopsis* and *Zea mays* database was conducted, with the RLM1 protein sequence used as a query sequence. A phylogenetic tree was generated by MEGA XI software; the unrooted tree was constructed according to the neighbor-joining method.

### Transcriptome Data Analysis

Total RNA was extracted from WT and *rlm1-D* plants in the 3rd-leaf stage. Seqhealth Technology Co., Ltd. (Wuhan, China) prepared the libraries and performed high-throughput sequencing. The reference genome sequence was downloaded from the NCBI database (PRJDB1747). KEGG enrichment analysis of differentially expressed genes was implemented by KOBAS software (version 2.1.1) with a corrected *p*-value cut-off of 0.05 to judge statistically significant enrichment.

### Y2H Assays

The full-length CDSs of RLM1 and OsMAPK10 were inserted into the *EcoRI* and *BamHI* sites of pGBKT7 (bait) and pGADT7 (prey), respectively, by an In-Fusion kit (Clontech, Japan). All the resulting constructs were verified by sequencing before being transformed into yeast strain AH109. The bait and prey plasmids were cotransformed into AH109 cells by an Ex-Yeast Transformation Kit (ZC135; ZOMANBIO, Beijing, China). The yeast colonies were transferred to plates containing SD/-Leu/-Trp and SD/-His/-Trp/-Leu/60 mM 3-AT media and then allowed to grow at 28°C for 3 days.

### Pull-Down Assays

Pull-Down assays were performed with GST-RLM1 and His-OsMAPK10. The RLM1 and OsMAPK10 coding sequences were cloned into pGEX6p-1 or pACYCDuet1 and purified with GST-Tag or His-Tag magnetic beads, respectively. GST-RLM1 and His-MAPK10 were subsequently incubated in binding buffer [20 mM sodium dihydrogen phosphate, 300 mM NaCl, 1 mM phenylmethylsulfonyl fluoride (PMSF), 1 mM dithiothreitol (DTT; pH 7.4)] for 2 h, after which binding buffer was used to wash the beads 5 times. Then, elution buffer [20 mM sodium dihydrogen phosphate, 300 mM NaCl, 500 mM imidazole (pH 7.4)] was applied to elute the proteins for subsequent western blot assays. Anti-RLM1 and anti-GST antibodies were utilized to detect the pulled down RLM1, and anti-His antibodies were utilized to detect the input.

### Split-LUC Complementation Assays

The RLM1 CDS was cloned into pCAMBIA1300-nLUC by an In-Fusion kit (Clontech, Japan), and the OsMAPK10 CDS was cloned into pCAMBIA1300-cLUC. The plasmid was sequenced for verification and then transformed into *Agrobacterium* strain GV3101. Four-week-old *N. benthamiana* plants were infiltrated by the transformed *Agrobacterium* cells and incubated at 23°C under dark conditions for 2 days; pCAMBIA1300-nLUC with pCAMBIA1330-cLUC served as a blank control. The leaves were sprayed with D-luciferin potassium salt (Yeasen, Shanghai, China), and the fluorescence was detected by an IVIS Lumina LT series III instrument (PerkinElmer).

### Y1H Assays

The full-length CDS of RLM1 was inserted into the *EcoRI* and *BamHI* sites of pGADT7 by an In-Fusion kit (Clontech). The MYB1AT motif from the *OsCAD2* promoter was inserted (as three tandem repeats) into pHis2 at both the *EcoRI* and *SacI* cloning sites. The plasmids were subsequently transformed into yeast strain Y187 by an Ex-Yeast Transformation Kit. The yeast colonies were transferred to plates containing SD/-Trp/-Leu and SD/-His/-Trp/-Leu media supplemented with 60 mM 3-AT and allowed to grow at 28°C for 3 days.

### EMSA Assays

The direct binding of RLM1 to the *OsCAD2* promoter was detected using an EMSA kit (GS009, Beyotime, Shanghai, China), following the manufacturer’s protocol, in conjunction with probes. The CDS of *RLM1* was cloned into the *Escherichia coli* expression vector pGEX6p-1 with a GST tag at the *BamHI* and *EcoRI* sites. The resulting plasmid was then transformed into the *E. coli* Rosetta strain and induced with 0.8 mM isopropyl β-D-1-thiogalactopyranoside (IPTG). Recombinant GST-RLM1 was purified using GST purification magnetic beads (abs9902, Absin, Shanghai China) according to the manufacturer’s protocol. The biotin-labeled oligonucleotide probes were synthesized and labeled by Shanghai Sangon Company (Shanghai, China).

### TF-Centered Y1H Assays

A 7 bp random motif library from yeast strain Y187 was purchased from Nanjing Ruiyuan Biotechnology Co., Ltd. (Nanjing, China). A TF-centered Y1H assay was performed as previously described (Ji et al., 2018). The yeast motif library was incubated overnight, after which an Ex-Yeast Transformation Kit was utilized to generate competent cells. Then, 35 μg of pGADT7-RLM1 was transformed into competent yeast library cells. The yeast was transferred to plates of SD/-His/-Trp/-Leu media supplemented with 60 mM 3-AT and allowed to grow at 28°C for 5 days. Monoclonal colonies were selected for sequencing, and random motif sequences between “GGG” and “CCC” (the *SmaI* site) were screened. The insertion sequences were analyzed using PLACE^2^ and PlantCARE^3^ to identify whether they were known motifs.

### Transactivation Activity Assay in Yeast

**Figure 1:**
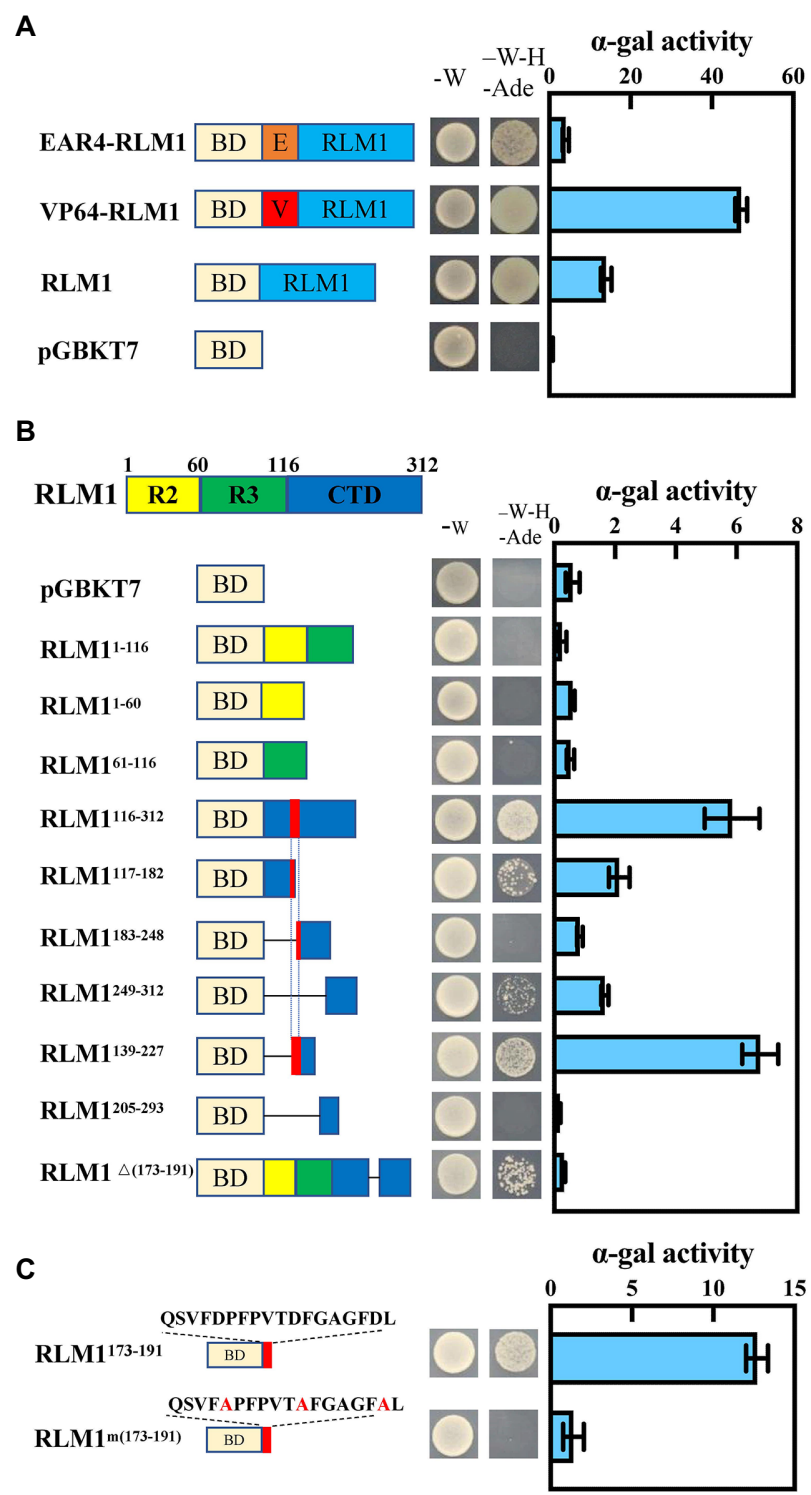
RLM1 acts as a transcriptional activator. **(A)** Transactivation analysis of RLM1, VP64-RLM1, and EAR4-RLM1 *via* a yeast assay. VP64 and EAR4 are an exogenous activation domain and an exogenous repression module, respectively. pGBKT7 alone was used as a negative control. Plate auxotroph and α-galactosidase assay showing the transcriptional activation of each protein. The bars represent the means ± SDs of three independent experiments. α-gal, α-galactosidase; -W, SD/-Trp; -W-H-Ade, SD/-Trp/-His/-Ade. **(B)** Mapping of the transactivation motif of RLM1 *via* a yeast assay. R2, aa 1–60; R3, aa 61–116; CTD, aa 117–312. Plate auxotroph and α-galactosidase assay showing the transcriptional activation of each protein. The bars represent the means ± SDs of three independent experiments. α-gal, α-galactosidase; pGBKT7, GAL4 DNA-binding domain; -W, SD; -W-H-Ade, SD/-Trp/-His/-Ade. **(C)** Transactivation analysis of the C-terminal motif QSVFDPFPVTDFGAGFDL *via* a yeast assay. BD represents the GAL4 DNA-binding domain. Mutations introduced into the C-terminal motif are indicated by the red font. Plate auxotroph and α-galactosidase assay showing the transcriptional activation by each protein. The bars represent the means ± SDs of three independent experiments. α-gal, α-galactosidase; -W, SD/-Trp; -W-H-Ade, SD/-Trp/-His/-Ade.

VP64 (encoding an exogenous activation domain) or EAR4 (encoding an exogenous repressor domain) was fused to the CDS of RLM1, which was then inserted into pGBKT7, and an Ex-Yeast Transformation Kit was used to transform these vectors into yeast strain AH109; the resulting yeast were then cultured on SD/-Trp media. Then, single colonies were selected and cultured on SD/-Ade-Trp-His media at 28°C for 3 days. The α-gal enzyme activity was assayed according to the Yeast Protocols Handbook (No. PT3024-1, Clontech). To finely map the active domain of OsRLM1, we divided the protein into different parts, as shown in Figure 1, and measured the α-gal enzyme activity.

### Transactivation Assay in Rice Protoplast

The promoter of *OsCAD2*, which was defined as the region 2 kb upstream from the transcription start site, was cloned and inserted into pGreenII-0800-Luc *via* the In-Fusion strategy to generate the corresponding reporter vector proOsCAD2-0800. The *RLM1* and *OsMAPK10* ORFs were cloned and inserted into pCAMBIA1307 to generate effector constructs. The resulting plasmids were transformed into rice protoplasts and cultured for 16 h. pCAMBIA1307 with pOsCAD2-0800 was used as a control. The LUC/REN ratio was determined by a Dual-Luciferase Reporter Assay System (Vazyme, DL101-01) according to the manufacturer’s instructions.

### *In vitro* Phosphorylation Assay

RLM1 phosphorylation by OsMAPK10 was performed with purified GST-RLM1 and His-OsMAPK10. The proteins were incubated in kinase buffer [50 mM hydroxyethyl piperazineethanesulfonic acid (HEPES; pH 7.4), 10 mM MnCl_2_, 1 mM DTT, 30 μM ATP] at 30°C for 30 min. The reaction was stopped, electrophoresis was performed on an SDS-PAGE gel (6% SDS, 5 μM Phos-tag, 10 μM MnCl_2_), and RLM1 was detected with an anti-GST antibody.

### Statistical Analysis

The experiments were repeated at least three times. The means with standard deviation are shown in the figures. Significant differences were determined by Student’s *t* test, and are marked with asterisks (^*^p < 0.05; ^**^p < 0.01; ^***^p < 0.001; ^****^p < 0.0001).

### Primer and Gene Sequences

The primers used are shown in Supplementary Table S3.

## Results

### Identification of *rlm1-D*

**Figure 2:**
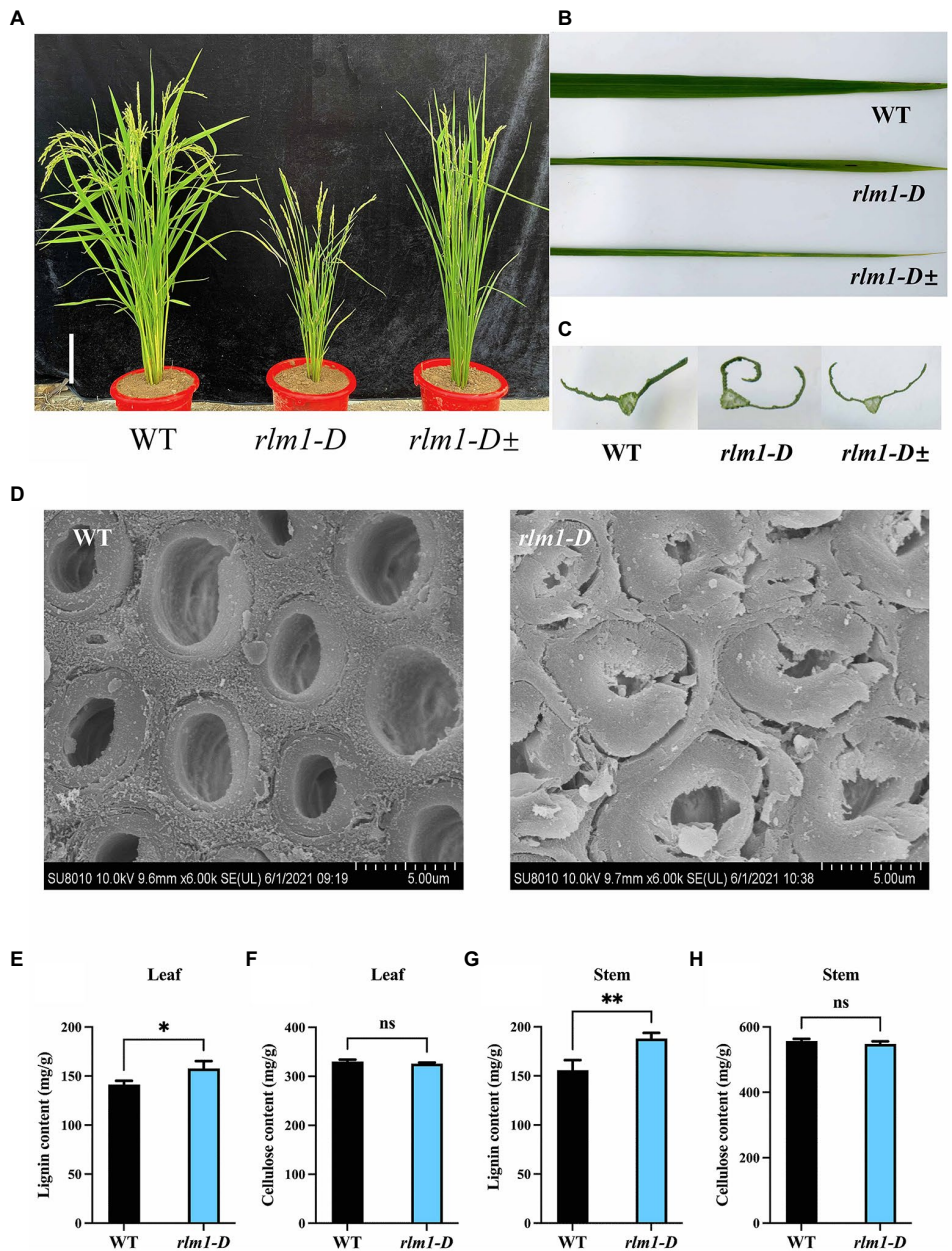
Clarification of the *rlm1-D* phenotype. **(A)** Phenotype comparisons of WT, *rlm1-D*, and *rlm1* heterozygous (*rlm1-D*±) plants at the heading stage. Bar = 5 cm. **(B)** Flag leaf comparison of the plants shown **(A)**. **(C)** Flag leaf cross-section comparisons of the plants shown in **(A)**. **(D)** SEM observations of sclerenchyma cell walls in the internodes from the 3-month-old WT and *rlm1-D* plants. Bar = 5 μm. **(E)** Lignin content measurements in WT and *rlm1-D* leaf tissue. **(F)** Cellulose content measurements in WT and *rlm1-D* leaf tissue. **(G)** Lignin content measurements in the internodes of the 3-month-old WT and *rlm1-D* plants. **(H)** Cellulose content measurements in secondary stem tissues of WT and *rlm1-D* plants. *p < 0.05, **p < 0.01 (Student’s *t*-test).

To explore the regulatory factors controlling leaf morphogenesis in rice, a rolling-leaf mutant, named *rlm1-D*, was obtained from a rice T-DNA mutant library (Wan et al., 2009). The *rlm1-D* presented dwarfism, rolling leaves, and a dark leaf color at the tillering stage (Figures 2A–C). In particular, owing to the excessive rolling of its leaves, the *rlm1-D* mutant did not produce seeds normally. There were three phenotypes of the *rlm1-D* heterozygous offspring: excessive-rolling, half-rolling, and wild-type like (WT-like) phenotypes, with which the corresponding segregation ratio was 1/2/1 (Supplementary Figure S1C). This suggests that *rlm1-D* may be controlled by a pair of dominant nuclear genes and that the *rlm1-D* gene may have a dose-dependent effect on leaf rolling.

To observe the cytological changes of *rlm1-D*, paraffin sectioning demonstrated that the size and number of bulliform cells in *rlm1-D* were significantly reduced compared to those in the WT (Supplementary Figures S1D,E). Since the *rlm1-D* plants exhibited a rolling-leaf phenotype accompanied by a dwarf-type phenotype, the features of *rlm1-D* were similar to those of the *roc8* and *rl14* mutants (Fang et al., 2012; Sun et al., 2020). Thus, we determined the leaf structure of *rlm1-D* and the WT using scanning electron microscopy (SEM). SEM results revealed that sclerenchyma cell wall (SCW) deposition was abnormal in *rlm1-D* internode tissues compared to WT tissues (Figure 2D). Therefore, we measured the contents of lignin and cellulose in *rlm1-D* and WT leaf and stem tissues. The results showed that the lignin contents of the stems and leaves of *rlm1-D* were significantly higher than those of the WT (Figures 2E,G). However, the cellulose content in *rlm1-D* was comparable to that in the WT (Figures 2F,H). The results of the abovementioned experiments indicated that abnormal SCWs in *rlm1-D* were perhaps the main factor responsible for leaf rolling. The abnormal SCWs in *rlm1-D* perhaps influenced water transport and indirectly regulated the size of bulliform cells, which promoted leaf rolling in *rlm1-D,* similar as *rl14* mutant (Fang et al., 2012).

### Map-Based Cloning of *RLM1*

**Figure 3:**
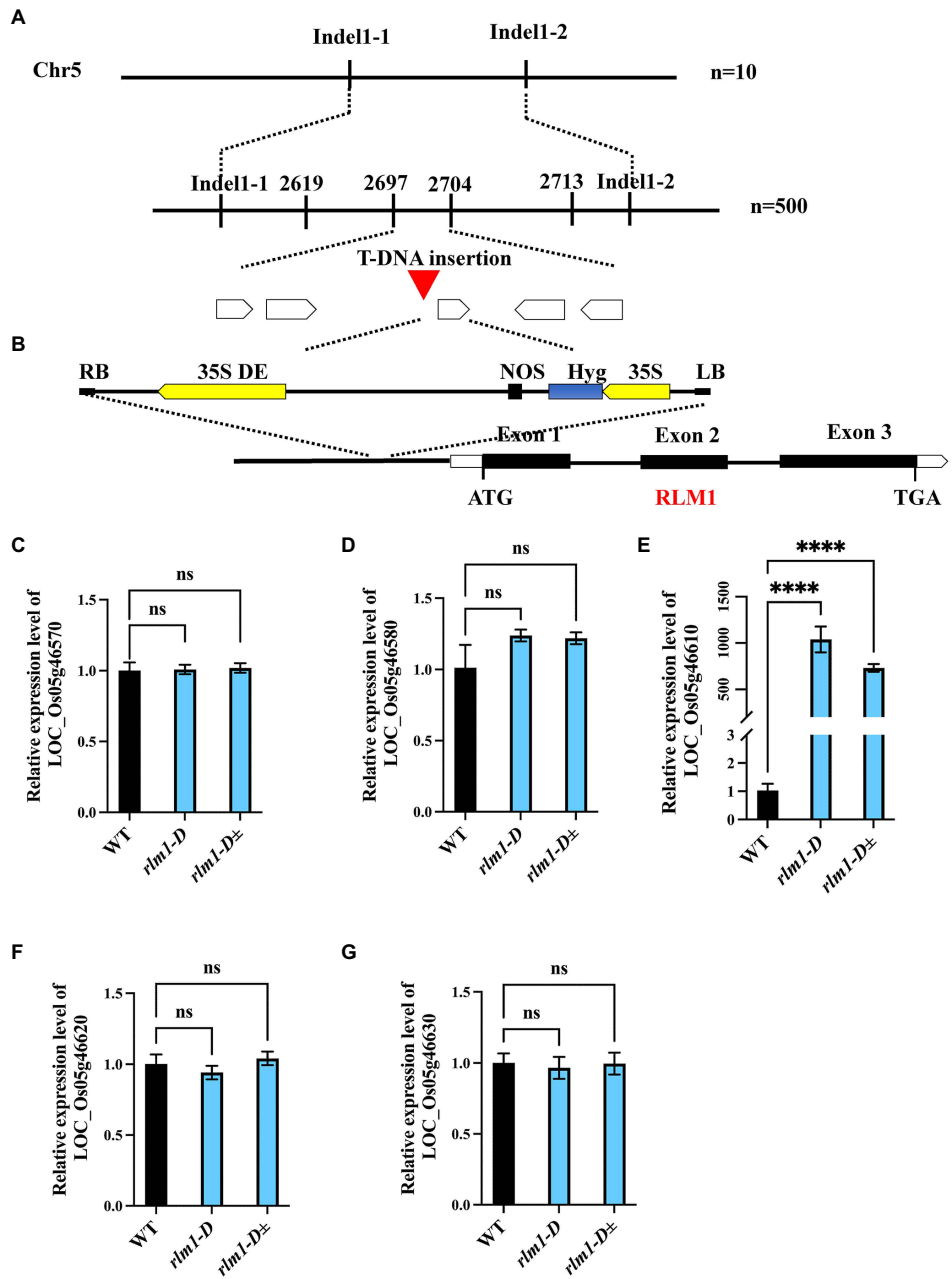
Map-based cloning of RLM1. **(A)** The RLM1 locus was mapped to a 2.5 cM interval on chromosome 5, between Indel markers Indel1-1 and Indel1-2. The location of the RLM1 locus was narrowed to a 10 kb region between insertion–deletion (InDel) markers 2,697 and 2,704 *via* 500 F2 WT-like plants. **(B)** The T-DNA insert site is located upstream of LOC_Os05g46610. The structure of RLM1 is shown. ATG and TGA, start and stop codons, respectively. Boxes, exons; lines between black boxes, introns. **(C–G)** Expression analysis of LOC_Os05g46570, LOC_Os05g46580, LOC_Os05g46570610, LOC_Os05g46620, and LOC_Os05g46630 in WT, *rlm1-D*, and heterozygous (*rlm1-D*±) plants. ****p < 0.0001 (Student’s *t*-test).

To clone *RLM1*, a genetic mapping population was generated by a cross between *rlm1-D* heterozygous plants and the rice *indica* cultivar *Dular*. *RLM1* was preliminarily mapped to a 25.2–27.72 cM range on chromosome 5. Further fine mapping narrowed *RLM1* to a 100 kb region (Figure 3A). Sequence analysis revealed a T-DNA insertion 750 bp upstream of the start codon (ATG) of LOC_Os05g46610 (Figure 3B). We speculated that the CaMV35S promoter in the T-DNA region activated LOC_Os05g46610 expression. To confirm this hypothesis, a quantitative reverse transcription PCR (qRT-PCR) experiment was performed to judge the expression levels of LOC_Os05g46570, LOC_Os05g46580, LOC_Os05g46610, LOC_Os05g46620, and LOC_Os05g46630 in *rlm1-D*, *rlm1-D* heterozygous plants and WT plants. The results demonstrated that the expression levels of the abovementioned genes (except for LOC_Os05g46610) in *rlm1-D* were comparable to those in the WT. The LOC_Os05g46610 expression level in *rlm1-D* increased by ~1,000-fold. Additionally, the expression level of LOC_Os05g46610 in the *rlm1-D* heterozygous plant increased to a level that was ~800-fold greater (Figures 3C–G). Overall, the map-based cloning and qRT-PCR analysis indicated that LOC_Os05g46610 was a candidate gene of *RLM1*.

**Figure 4:**
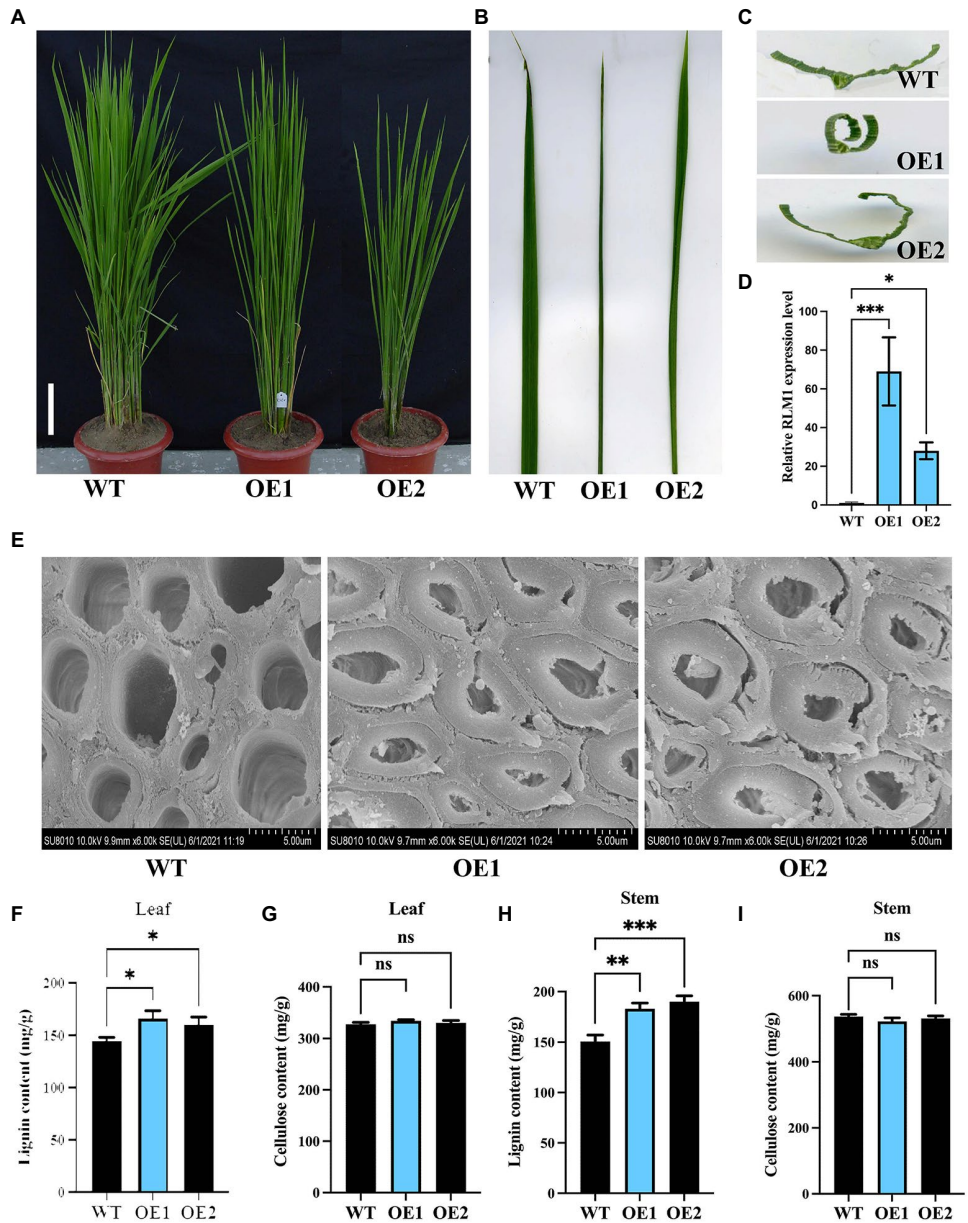
Analysis of RLM1 overexpression in the WT background. **(A)** Comparison of WT and RLM1 overexpression plants (OE1, OE2) at the late-tiller stage. Bar = 5 cm. **(B)** Flag leaf comparisons of the plants shown in **(A)**. **(C)** Flag leaf cross-section comparisons of the plants shown in **(A)**. **(D)**. RLM1 expression level analysis of WT and RLM1-overexpressing plants (OE1, OE2). **(E)** SEM observations of sclerenchyma cell walls in the internodes from the 3-month-old WT, OE1, and OE2 plants. Bar = 5 μm. **(F)** Lignin content measurements in WT, OE1, and OE2 leaf tissues. **(G)** Cellulose content measurements in WT, OE1, and OE2 leaf tissue. **(H)** Lignin content measurements in the second internode tissues from 3-month-old WT, OE1, and OE2 plants. **(I)** Cellulose content measurements in the second internode tissues from 3-month-old WT, OE1, and OE2 plants. *p < 0.05, **p < 0.01, ***p < 0.001 (Student’s *t*-test).

To further verify that LOC_Os05g46610 is responsible for the *rlm1-D* phenotype, an overexpression experiment was conducted. *Agrobacterium tumefaciens* strain AGL1 containing the recombinant construct (LOC_Os05g46610-pCambia1302) was used to infect the *Nipponbare* calli. Fifteen lines among the T_1_ progeny revealed rolling-leaf and dwarf-type phenotypes (Figures 4A–C). qRT-PCR analysis showed that the degree of the leaf rolling among the overexpression lines was positively correlated with the expression level of LOC_Os05g46610 (Figure 4D). SEM experiments also demonstrated that SCW deposition was abnormal in RLM1-OE1 and RLM1-OE2 (Figure 4E). Lignin and cellulose content measurements also verified the increased lignin content and normal cellulose content in RLM1-OE1 and RLM1-OE2, the contents of which were similar to those of the *rlm1-D* mutant (Figures 4F–I). The results of the abovementioned experiments confirmed that ectopic expression of LOC_Os05g46610 was responsible for the rolling-leaf phenotype of *rlm1-D*.

### Bioinformatics and Molecular Characteristics of RLM1

BLAST analysis demonstrated that RLM1 encoded an R2R3 MYB transcription factor. R2R3 MYB transcription factors are major transcriptional regulators that mediate a variety of plant biological processes, including seed germination, drought tolerance, stomatal conductance, lateral root development, hormone biosynthesis, anthocyanin accumulation, and cuticle wax biosynthesis (Jiang and Rao, 2020). The function of RLM1, which encodes an R2R3 MYB transcription factor, is unknown. RLM1 contains three exons and two introns, and its predicted ORF was 936 bp and encoded 312 amino acids (aa). Protein motif analysis demonstrated that RLM1 contained an N-terminal domain (R2R3) and a C-terminal domain (CTD). The N-terminus likely mainly interacts with other proteins, and the C-terminus activates or inhibits downstream target genes (Meng et al., 2019).

The amino acid sequence of RLM1 is 55% homologous with that of AtMYB4 from *Arabidopsis* (Supplementary Figure S2). In *Arabidopsis*, AtMYB4 acts as a transcriptional repressor (Jin et al., 2000). To confirm whether RLM1 has transcriptional inhibitory activity, the constructs (RLM1-pGBKT7, VP64-RLM1-pGBKT7, EAR4-RLM1-pGBKT7) were expressed in yeast, respectively. The yeast colony status on the selected media (SD/−Ade-Trp-His) showed that the activation activity of VP64-RLM1-pGBKT7, RLM1-pGBKT7, pGBKT7, and EAR4-RLM1-pGBKT7 decreased successively (Figure 1A). The above results showed that, in contrast to AtMYB4, RLM1 mainly had a transcriptional activation activity.

To further study which region of RLM1 had activation activity, the full-length RLM1 sequence was divided into three regions: R2 (RLM1^1–60^), R3 (RLM1^61–116^), and CTD (RLM1^116–312^). Then, three different constructs (R2-pGBKT7, R3-pGBKT7 and CTD-pGBKT7) were expressed in yeast, respectively. Yeast activation assays revealed that R2-pGBKT7 and R3-pGBKT7 had no activation effect in yeast growing on selective media (SD/-Ade-Trp-His). However, CTD- pGBKT7 had a stronger activation effect on yeast growing on the selective medium (SD/-Ade-Trp-His; Figure 1B).

Next, we tested the activation activities of different CTD deletion constructs. Yeast activation experiments revealed that an 18 amino acid (aa) sequence (QSVFDPFPVTDFGAGFDL) was very important for the activation activity of RLM1. Partial mutation or full deletion of these 18 aa had a great impact on the activation activity of RLM1. Moreover, the 18 aa motif alone had strong transcriptional activation activity, similar to that of the full RLM1-pGBKT7. This 18 aa motif contains some acidic amino acids, and after these acidic amino acids were mutated (Glu/Asp replaced by Ala), the activation activity was no longer detected, indicating that the acidic amino acids of these 18 aa were very important for the activation activity of RLM1 (Figure 1C).

### Temporal and Spatial Expression Patterns of RLM1

To determine the tissue specificity of *RLM1, A. tumefaciens* strain containing proRLM1-1391Z construct were used to infect *Nipponbare* calli. β-Glucuronidase staining of T_1_ progeny revealed that RLM1 is mainly expressed in young seedlings. In particular, RLM1 was also specifically expressed in the stem, possibly at the site of the intermediate meristem at the mature stage, and RLM1 was not expressed in the leaf, panicle, or root tissue at the mature stage (Supplementary Figures S3A**–**H). The qRT-PCR experiment showed that *RLM1* was highly expressed at the young seedling stage and in stem tissue at the mature stage but was not expressed in the other tissues, which is generally consistent with the GUS staining assay results (Supplementary Figure S3I).

*RLM1* encodes an R2R3-type MYB transcription factor and may localize to the nucleus. To judge whether RLM1 is localized in the nucleus, a RLM1-pAN580 construct was transiently transformed into rice protoplasts. Confocal observations revealed that RLM1 indeed localized to the nucleus and acted as a transcription factor (Supplementary Figures S4A**–**D).

### Transcriptome Analysis of RLM1

To verify the regulatory pathways in which RLM1 is involved, RNA samples from 3^rd^-leaf stage WT and *rlm1-D* seedlings were collected. Then, high-throughput sequencing and analysis were performed by Seqhealth Technology Co., Ltd. (Wuhan, China). We obtained high-quality data (BioProject accession: PRJNA813008), with 97% of the reads mapped to the reference transcriptome. A total of 1,777 genes were differentially expressed in the *rlm1-D* plants, of which 973 genes were upregulated and 804 genes were downregulated, which is consistent with RLM1 acting as a transcriptional activator (at least three repeats, *p* ≤ 0.01; Supplementary Table S1). Kyoto Encyclopedia of Gene and Genomes (KEGG) analysis demonstrated that the upregulated genes were involved in the phenylpropanoid metabolic pathway and that the downregulated genes played a role in the biosynthesis of secondary metabolites (Supplementary Figures S5A,B). We selected 19 upregulated genes involved in the phenylpropanoid metabolic pathway. The gene expression levels were confirmed by qRT-PCR, and the results were generally consistent with the transcriptome data (Supplementary Figure S6A).

### RLM1 Target Binding Gene Analysis

To identify the target sites that may be directly regulated by RLM1, using transcription factor-centered (TF-centered) yeast one-hybrid (Y1H) technology, we screened the motifs of RLM1-binding target sites. A pHis2 yeast library containing 7 bp random fragments was constructed and screened by using RLM1-AD according to the mentioned methods (Ji et al., 2014). A total of 122 motifs were obtained to perform enrichment analysis,^4^ and we found that 20 motifs possibly bind to RLM1 (Supplementary Table S2). Selection on yeast screening media (SD/-Trp-Leu-His with 60 mM 3-AT) revealed that all 20 motifs could be recognized by RLM1 (Supplementary Figure S7). These motifs are mainly composed of cis-acting elements whose core sequence is “MYB1AT.” However, other cis-acting elements, such as *CAATBOX1* and *GATABOX*, were also found.

Combined with the transcriptome sequencing results, additional results showed that these motifs were present in the promoter of genes upregulated in the phenylpropanoid metabolic pathway. Because OsCCR1 and OsCAD2 showed higher expression in *rlm1-D* and its promoter contained an MYB1AT motif (Supplementary Figures S6B,C) and *OsCAD2* was responsible for rice lignin synthesis (Ookawa et al., 2014). We selected *OsCAD2* gene for further study.

**Figure 5:**
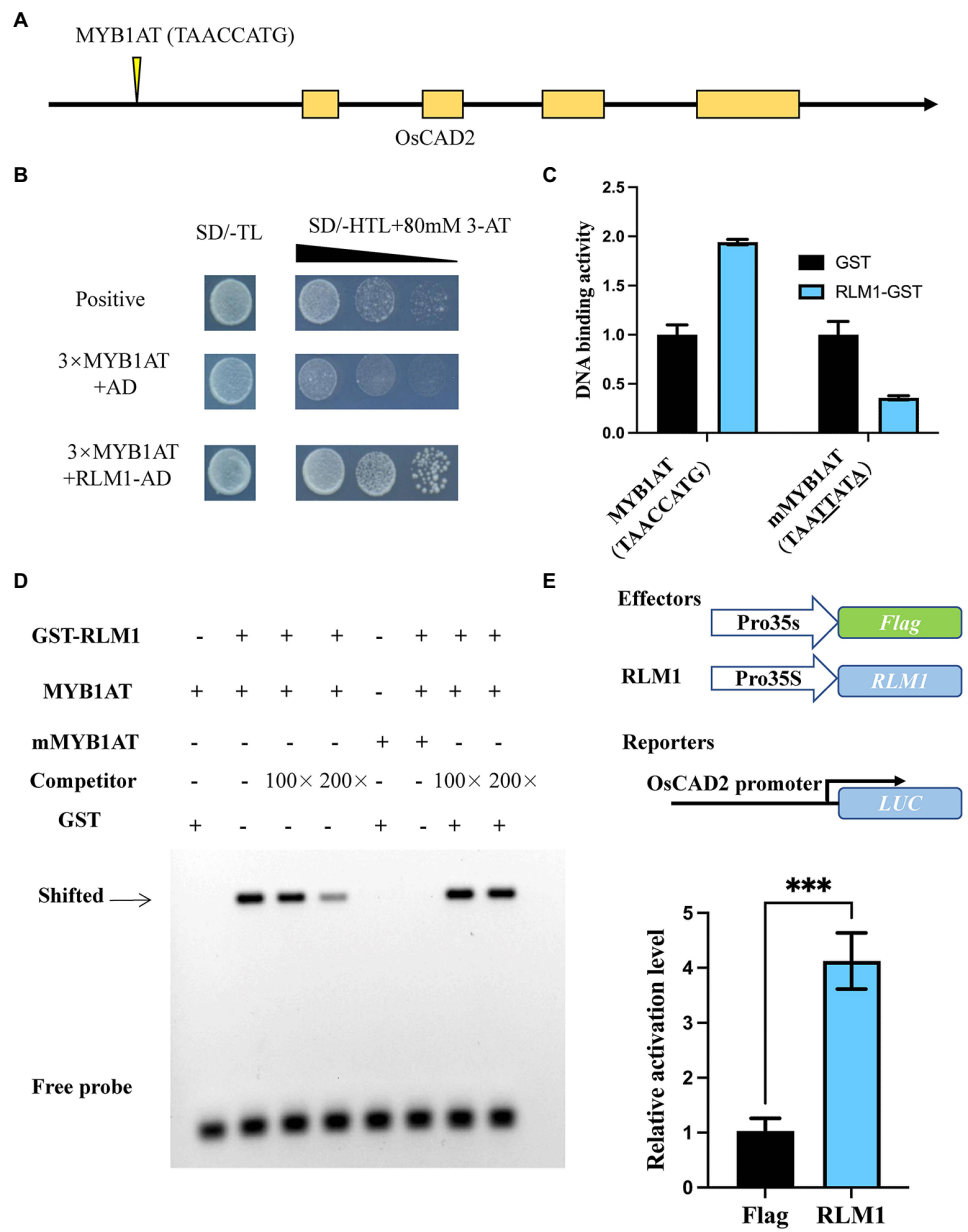
RLM1 binds to the motif of OsCAD2 and activates its expression. **(A)** The MYB1AT motif box is located approximately 1,931–1,938 bp upstream of the *OsCAD2* start codon. **(B)** A Y1H assay was used to test RLM1 binding to the MYB1AT motif. An AD-only vector was used as a control. **(C)** DNA-protein binding assay revealing that RLM1 binds to the MYB1AT motif in the *OsCAD2* promoter. MYB1AT, TAACCATG; mMYB1AT, TAATTATA. **(A)** Schematic diagram of *OsCAD2*. MYB1AT (TAACCATG) indicates the position in the *OsCAD2* promoter. **(B)** Y1H assays show that RLM1 can bind to the MYB1AT motif, compared with the binding of the negative and positive controls. **(C)** DNA-binding activity of each group. GST was used as a negative control. The data are presented as the means ± SDs (*n* = 3). **(D)** EMSA shows that the GST-RLM1 recombinant protein directly binds to the biotin-labeled probe of the *OsCAD2* promoter fragment. The signal in the RLM1 group and the MYB1AT motif in the *OsCAD2* promoter shifted, but no signal shift was found in the RLM1 or mutant MYB1AT group. The shifted signal became increasingly weaker and eventually was no longer detected when unlabeled competitive probes were added. MYB1AT, TAACCATG; mMYB1AT, TAATTATA. **(E)** RLM1 induces proOsCAD2::LUC signaling. LUC activity was detected in rice protoplasts. The expression of REN was used as an internal control. The LUC/REN ratio indicates the expression level of the promoter (*n* = 3). ^***^p < 0.001 (Student’s *t*-test).

Y1H assays confirmed that RLM1 could recognize and bind to the promoter of *OsCAD2* (Figures 5A,B). A DNA-binding assay was performed to detect whether RLM1 can bind to the *OsCAD2* promoter region *in vitro*. The results revealed that RLM1 fused to a glutathione S-transferase (GST) tag could bind to the MYB1AT motif, while the binding was significantly weakened in a mutant MYB1AT motif (TAACCATG to TAATTATA; Figure 5C). Next, EMSA experiment was used to judge the RLM1 binding to the promoter of OsCAD2. We incubated GST-RLM1 proteins together with a biotin-labeled probe. The protein-DNA complex showed an obvious band shift, but the mutant probe did not (Figure 5D). Thus, these results verified that RLM1 could bind to the *OsCAD2* promoter containing the MYB1AT motif. In addition, a luciferase (LUC) activity assay was used to determine whether RLM1 can activate *OsCAD2*. In rice protoplasts cotransfected with the effector and reporter vectors, the ratio of firefly LUC to Renilla luciferase (REN) of the effector OsCAD2 pro-LUC was fourfold higher than that of the empty vector control (Figure 5E).

### RLM1 Interaction Partners

**Figure 6:**
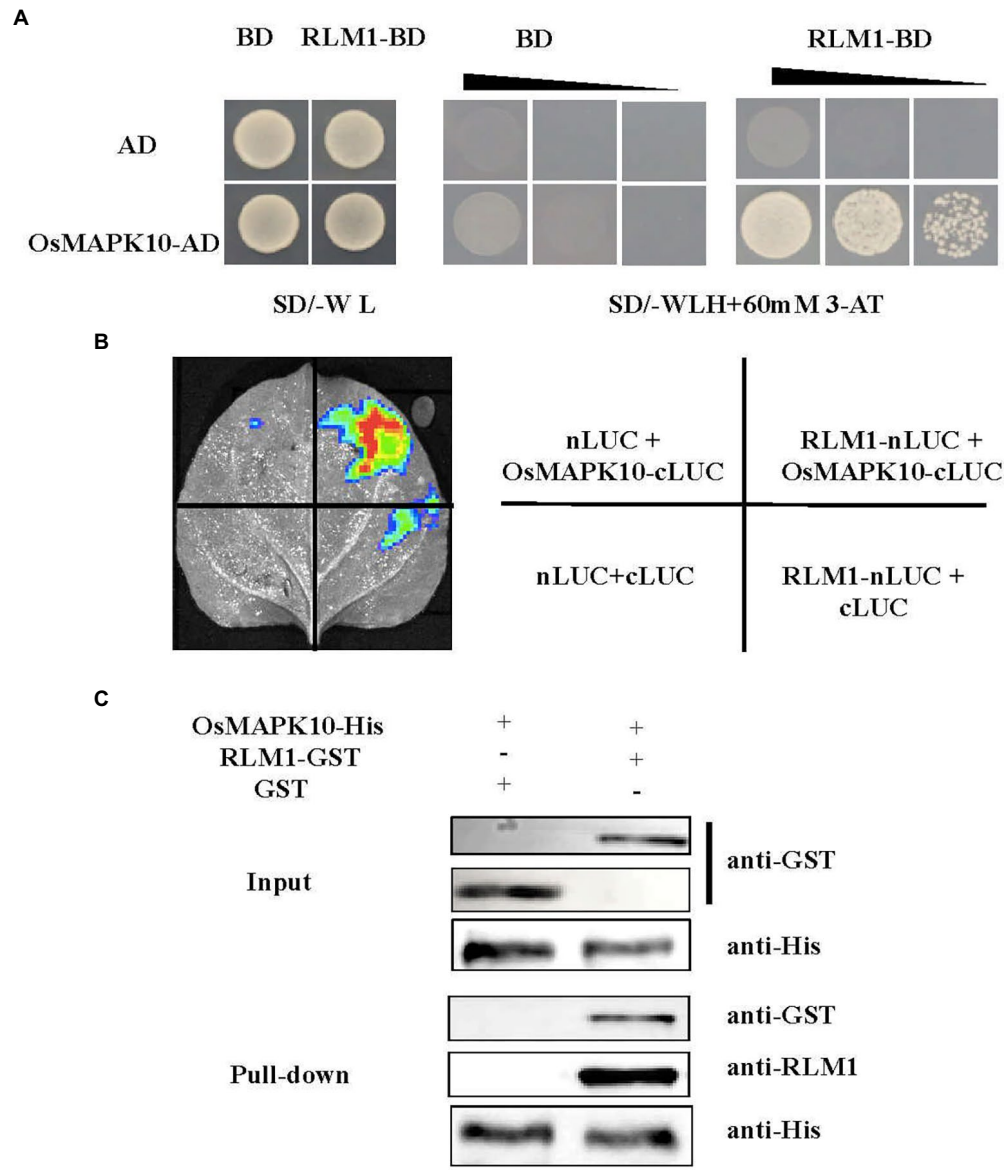
RLM1 physically interacts with OsMAPK10. **(A)** A Y2H interaction assay was performed between RLM1 and OsMAPK10. A pGADT7 vector (AD) was used as the negative control. RLM1 was fused to the pGBKT7 vector (RLM1-BD). OsMAPK10 proteins were fused to the pGADT7 vector (OsMAPK10-AD). **(B)** The full OsMAPK10 protein was fused to the pCAMBIA1300-cLUC vector (OsMAPK10-cLUC). The full RLM1 protein was fused to pCAMBIA1300-nLUC (RLM1-nLUC). Split-LUC complementation assay showing that RLM1 can interact with OsMAPK10 in cells of *N. benthamiana* leaves. The LUC signals were not detected in the corresponding negative controls. **(C)** Pull-Down assays of RLM1 with OsMAPK10 *in vitro*. RLM1-GST was incubated together with OsMAPK10-His beads, and GST proteins were used as controls. Western bolt was detected *via* anti-GST, anti-RLM1, and anti-his antibodies, respectively. Compared with the input and Pull-Down band results, the results showed that GST did not interact with OsMAPK10, but RLM1 interacted with OsMAPK10.

To clarify the regulatory network of RLM1, a yeast library screening assay was conducted. First, a self-activation test of RLM1-pGBKT7 was performed. We found that RLM1-pGBKT7 could not grow in selective media (SD/-Trp-His-Leu + 60 mM 3-AT). Then, the yeast screening library assay was carried out with a concentration of 60 mM 3-AT, and multiple clones were obtained by screening and sequencing. OsMAPK10 was repeated at least five times (Figure 6A). Through the RiceXPro database, we found that OsMAPK10 had a similar expression pattern as RLM1 (Supplementary Figures S8A,B). We subsequently carried out a follow-up study of OsMAPK10.

Three experiments were applied to verify the relationship between OsMAPK10 and RLM1. First, the full-length OsMAPK10 coding sequence was amplified and fused to pGADT7, and the yeast two-hybrid (Y2H) assay showed that OsMAPK10 interacted with RLM1-pGBKT7 in selective media (SD/-Trp-His-Leu + 60 mM 3AT; Figure 6A). Second, we used the split-LUC complementation experiment to judge the interaction of both. The pCAMBIA1300-nLUC (RLM1-nLUC), and pCAMBIA1300-cLUC (OsMAPK10-cLUC) were transformed into *Agrobacterium* GV3101, respectively. The different combinations were injected into tobacco, and LUC fluorescence was detected. The results suggested that RLM1-nLUC and MAPK10-cLUC could interact, causing LUC to be expressed and fluoresce (Figure 6B). Third, the pull-down assay also confirmed the interaction of OsMAPK10 and RLM1. The GST-RLM1 and GST proteins were incubated with beads bound to His-MAPK10, washed several times after incubation, denatured, detected *via* western blotting, and hybridized to anti-GST and anti-RLM1 antibodies. We found that GST-RLM1 proteins could be detected, but GST proteins could not be (Figure 6C). Taken together, the results of the three above mentioned experiments proved that RLM1 interacts with OsMAPK10.

### Generation of OsMAPK10 Knockdown Lines and Overexpression Lines

To further study whether *OsMAPK1*0 participates in leaf morphogenesis, *OsMAPK10* knockout lines were created *via* gene-editing technology. The mutation of knockout line 1 (*OsMAPK10*-*m1*) occurred in the second exon, and 10 bp was deleted. The mutation of knockout line 2 (*OsMAPK10*-*m2*) occurred in the second exon, and 1 bp was deleted (Supplementary Figure S9A). Both mutations resulted in frameshift mutations (Supplementary Figure S9B). qRT-PCR analysis demonstrated that the expression level of *OsMAPK10* reached a lower level than it did in the WT (Supplementary Figure S9D). The *OsMAPK10*-*m1* and *OsMAPK10*-*m2* lines were considered knockdown mutants. The *OsMAPK10*-*m1* and *OsMAPK10*-*m2* lines had a dwarf-type phenotype in the field (Supplementary Figure S9C). There were lower lignin contents in the leaves and stems in the *OsMAPK10*-*m1* and *OsMAPK10*-*m2* lines compared to the WT (Supplementary Figures S9E**–**H). Overexpression of *OsMAPK10* driven by CaMV35S resulted in increased lignin contents in the leaves and stems of OE1 and OE2, similar to those of *rlm1-D* (Supplementary Figures S10A**–**G). The knockdown and overexpression experiments of *OsMAPK10* showed that OsMAPK10 may directly participate in SCW deposition.

### Relationship Between OsMAPK10 and RLM1

Three experiments were performed to study the relationship between OsMAPK10 and RLM1. MAPK family proteins mainly function by phosphorylating interacting partners. To judge whether OsMAPK10 phosphorylates RLM1, His-OsMAPK10 and GST-RLM1 fusion constructs were transformed into the *E. coli* Rosetta strain (DE3). Both fusion proteins were incubated and hybridized with GST antibody with or without the addition of a Phos-tag. The results demonstrated that when Phos-tag was added to the SDS-PAGE gel, the hybridization band was lagged without the addition of the Phos-tag (Supplementary Figure S11A). These results indicated that RLM1 could be phosphorylated by OsMAPK10. A LUC activity assay was applied to detect whether OsMAPK10 can facilitate RLM1 activation of downstream target genes. In rice protoplasts cotransfected with the effector and reporter vectors, the ratio of LUC to REN of the effector pOsCAD2-LUC was threefold higher than that of the empty vector control. While OsMAPK10 and RLM1 were transferred into rice protoplast together, the ratio of LUC to REN of the effector pOsCAD2-LUC was sixfold higher than that of the empty vector control (Supplementary Figure S11B). The above experiments preliminarily suggested that RLM1 is phosphorylated by OsMAPK10 and that phosphorylated RLM1 may activate the expression of downstream target genes.

### RLM1 Knockdown Lines Have a Potential Effect on Rice Yield

**Figure 7:**
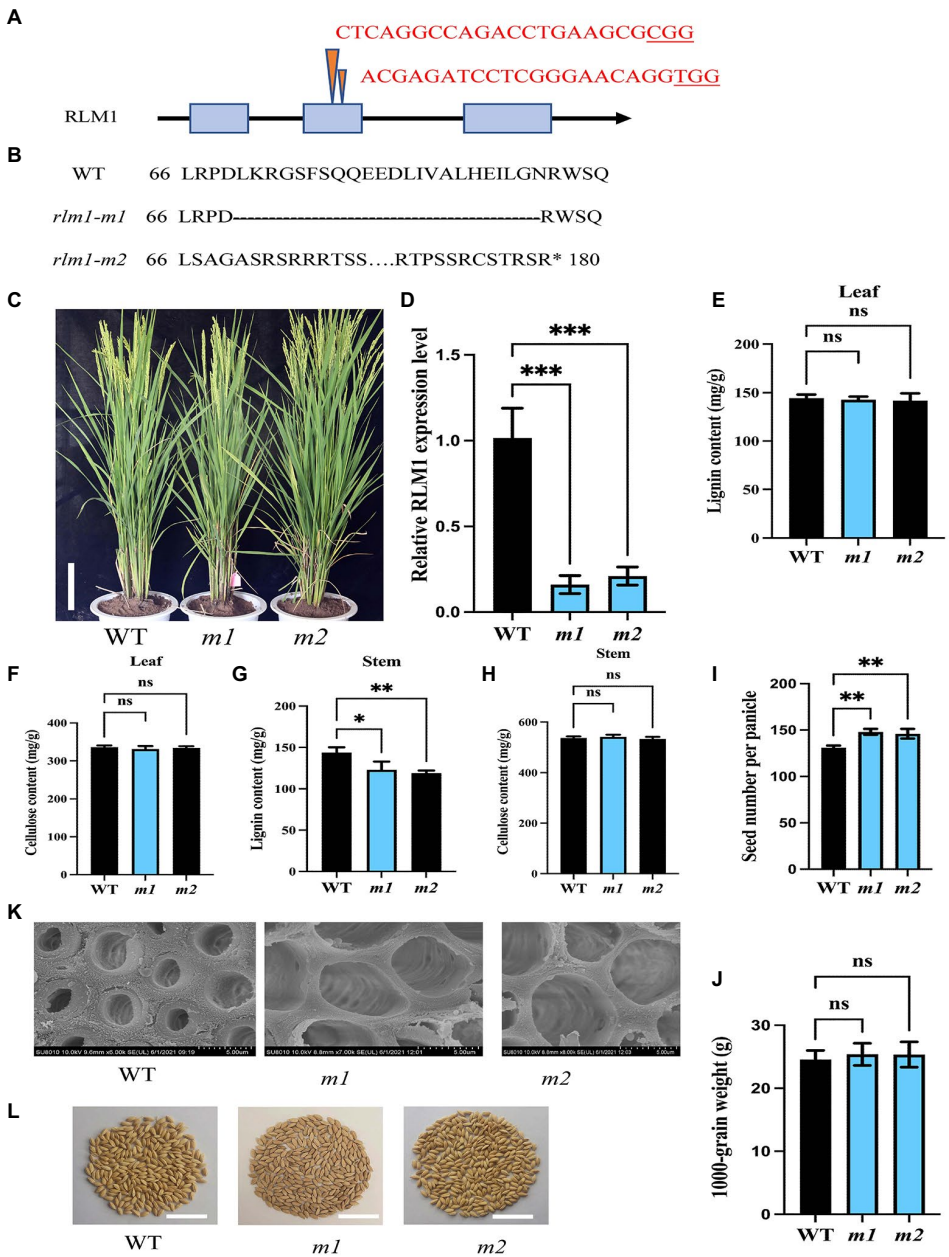
CRISPR-Cas9-induced mutations in RLM1. **(A)** Schematic map of the genomic region of RLM1 and the sgRNA target site. The arrow shows the sgRNA target site of the RLM1 genomic sequence, and the PAM motif (NGG) is shown in red. The blue boxes represent RLM1 exons, and the black lines indicate intron sequences. **(B)** Amino acid alignment surrounding the sgRNA target region showing the predicted peptide sequence of the WT and mutant alleles. The site of the predicted frame shifted sequence is underlined, and new stop codons are indicated by asterisks. **(C)** Phenotypes of the WT and homozygous mutants *rlm1-m1* and indicated *rlm1-m2* at the heading stage (Bar = 5 cm). **(D)** qRT-PCR analysis showing RLM1 expression levels in WT plants and *rlm1-m1* and *rlm1-m2* homozygous mutant plants. *OsActin1* was selected as an internal reference. **(E)** Lignin content measurements in WT, *rlm1-m1,* and *rlm1-m2* leaf tissue. **(F)** Cellulose content measurements in WT, *rlm1-m1,* and *rlm1-m2* leaf tissue. **(G)** Lignin content measurements in the second internodes from 3-month-old WT, *rlm1-m1*, and *rlm1-m2* plants. **(H)** Cellulose content measurements in the second internodes from 3-month-old WT, *rlm1-m1*, and *rlm1-m2* plants. **(I)** Comparison of seed number per panicle among WT, *rlm1-m1*, and *rlm1-m2* plants (*n* = 15). **(K)** SEM observations of sclerenchyma cell walls in the internodes from the 3-month-old WT, *rlm1-m1,* and *rlm1-m2 plants*. Bar = 5 μm. **(J)** Comparison of 1,000-grain weight among WT, *rlm1-m1*, and *rlm1-m2* plants (*n* = 15). **(L)** Seeds per panicle of WT, *rlm1-m1,* and *rlm1-m2*. Bar = 5 cm. The bars represent the SDs of the means. The student’s *t*-test was performed to determine the significance: *p < 0.05, **p < 0.01, ***p < 0.001 (Student’s *t*-test).

To explore the function of *RLM1*, two *RLM1*-deletion mutant lines (*rlm1-m1* and *rlm1-m2*) were generated using CRISPR editing technology. The *rlm1-m1* line and *rlm1-m2* line contained a 69 bp deletion in the second exon of RLM1 (from 199 to 267 bp) and a 1 bp deletion in the second exon of RLM1 (at 203 bp), respectively (Figures 7A,B). The qRT-PCR of *rlm1-m1* and *rlm1-m2* plants showed that the expression level of *RLM1* was reduced significantly in the *rlm1-m1* and *rlm1-m2* plants, which indicated that both plants were knockdown mutants (Figure 7D). The leaf morphology phenotypes of the two knockdown lines were similar to that of the WT (Figure 7C). Because RLM1 was expressed in the stems only at maturity, SEM observation was applied, which revealed that the SCWs in the *rlm1-m1* and *rlm1-m2* stems were significantly thinner than those in the WT (Figure 7K). The result in the *rlm1-m1* and *rlm1-m2* stems was just contrary to the cytological observation of *RLM1* overexpression lines. The lignin content of the *rlm1-m1* and *rlm1-m2* stems also decreased slightly compared to that of the WT (Figure 7G). However, the cellulose and lignin contents of the leaves of the *rlm1-m1* and *rlm1-m2* plants did not change significantly (Figures 7E,F,H). Moreover, the *rlm1-m1* and *rlm1-m2* had more grains per panicle (Figure 7I), and the yield per plant increased by 11% compared with that of the WT (Figure 7L), whereas the 1,000 grain weight in *rlm1-m1* and *rlm1-m2* were similar to WT (Figure 7J).

## Discussion

Rice yield is mainly determined by leaf morphology and plant type. At present, many genes controlling leaf morphogenesis have been identified. However, how to understand the regulation relationship of leaf morphogenesis and plant type remains unclear. In this study, by screening a T-DNA insertion mutant population, we obtained a rolling-leaf mutant, *rlm1-D*, and cloned the *RLM1* gene *via* the map-based cloning method. *RLM1* encodes a typical R2R3 MYB transcription factor, and we proposed a preliminary a regulatory network based on cytological, biochemical, and genetic evidence. First, most of the reported genes, such as *LC2* (Zhao et al., 2010), *OsHB1* (Itoh et al., 2008), *ACL1/ACL2* (Li et al., 2010), *BRD1* (Hong et al., 2002), *CLD1/SRL1* (Li et al., 2017), *YAB1* (Dai et al., 2007), *OsCSLD4/NRL1* (Hu et al., 2010), and *OsAGO7* (Shi et al., 2007), directly regulate the development of bulliform cells and promote leaf rolling. A series of experiments, such as those involving paraffin sectioning, SEM observations, and measurements of cellulose and lignin contents, confirmed that the abnormal SCW disposition in *rlm1-D* plants was the direct cause of the *rlm1-D* rolling-leaf and dwarf-type phenotypes, and the reduction in the number and size of bulliform cells in *rlm1-D* was a secondary effect; these mechanisms were different from that governing the rolling-leaf phenotype. This mechanism controlling leaf morphology in *rlm1-D* was similar to that involving *RL14* (Fang et al., 2012) and *ROC8* (Sun et al., 2020). Second, multiple conclusions supported the *rlm1-D* was caused by the ectopic expression of *RLM1*. The expression level analysis showed that *RLM1* is highly expressed in all parts of *rlm1-D* mutant (Supplementary Figure S12). Overexpression of *RLM1* in *Nipponbare* derived from CaMV35S reproduces the rolling-leaf and dwarf-type phenotype, similar to the *rlm1-D* mutant phenotype. There was a dose-dependent effect on RLM1 expression due to heterologous activation of RLM1. Third, the biochemical analysis of RLM1 also confirmed that RLM1 had transcriptional activity, and this activity was determined by the CTD motif. Further experiments involving the deletion of the CTD motif determined that 18 aa was important for RLM1 transcriptional activity. Fourth, transcriptome analysis of *rlm1-D* and WT plants confirmed that RLM1 regulated the phenylpropanoid pathway. qRT-PCR, Y1H assay, and electrophoretic mobility shift assay (EMSA) experiments determined that RLM1 targets the promoter of OsCAD2 responsible for lignin synthesis by binding the MYB1AT motif. Fifth, Expression pattern analysis at the young seedling and mature stages suggested that RLM1 was mainly expressed in young seedlings and in mature stems, not in the other tissues of mature plants. The temporal and spatial expression specificity of RLM1 determines the role of RLM1 in leaf morphology and plant type. Totally, the above five aspects support that *RLM1* influences SCW deposition by regulating lignin synthesis in leaves and stems in rice.

The components of the cell wall include cellulose, hemicellulose, and lignin, which provide support and defensive ability to plants. Together, the components of the cell wall also play the most important role in terms of biomass energy, degradation, and the transformation of straw. Therefore, the pyramidal hierarchy of SCW disposition regulation was determined in model plant species. NACs are the top-layer transcription factors for SCW deposition, and MYB transcription factors constitute the most important hub (Wang et al., 2021). Many MYBs in rice have been found to be involved in regulating the development of the SCW. *OsMYB103L* mediates cellulose biosynthesis and secondary wall formation in leaves and stems mainly by directly binding the promoters of *CESA4*, *CESA7*, *CESA*9, and *BC1* and regulating their expression (Ye et al., 2015). NAC29/31 directly regulates *OsMYB61*, which in turn activates CESA expression (Ye et al., 2018). However, the function of *RLM1* was different from that previously reported for *OsMYB61* and *OsMYB103L*. We found that *RLM1* regulates SCW deposition by regulating lignin synthesis. Lignin synthesis is specifically initiated in the cells that form SCWs, providing mechanical support for the upright growth of plants, helping to establish long-distance transport channels for water and materials, and strengthening the stress resistance barrier of plants. Notably, AtMYB4 is highly homologous to RLM1. AtMYB4 was shown to regulate the accumulation of the UV protectant compound sinapoyl malate through its ability to repress the transcription of the gene encoding the phenylpropanoid pathway enzyme cinnamate 4-hydroxylase (Jin et al., 2000). RLM1 acts as an activator, and AtMYB4 acts as a repressor. The opposite function of the two depends on 18 aa.

As components of complexes, MAPKs and MYBs participate in different biological development processes in plants. Mitogen-activated protein kinase 6 negatively regulates SCW biosynthesis by modulating MYB46 protein stability in *Arabidopsis* (Im et al., 2021). MYB75 phosphorylation by MPK4 is required for light-induced anthocyanin accumulation in *Arabidopsis* (Li et al., 2016). However, the biological functions of MAPK and MYB complexes remain unknown in rice. OsMAPK10 regulates RLM1 based on the following considerations. Like that of RLM1, the expression of OsMAPK10 occurs predominantly in the stems. Y2H, split-LUC complementation, and pull-down assays confirmed the interaction between OsMAPK10 and RLM1. OsMAPK10 can phosphorylate RLM1. LUC activity assays revealed that OsMAPK10 facilitates the activation of downstream target genes by RLM1. We will study the specific phosphorylation sites in the future. Nevertheless, the knockdown lines of OsMAPK10 displayed a semi dwarf-type phenotype, and knockdown lines of RLM1 showed a normal-leaf phenotype, indicating that OsMAPK10 is perhaps involved in other plant development processes. The above mentioned findings suggest that a OsMAPK10-RLM1-OsCAD2 regulatory network controls leaf morphogenesis and plant type. However, the RLM1 phosphorylation sites modified by OsMAPK10 still need to be identified.

### Potential Application of RLM1

Temporal and spatial expression specificity of RLM1 indicates that *RLM1* has potential application in production. In the *RLM1* knockout lines, the morphological development of the leaves is not affected, but the SCW deposition of stems is modified. The aperture around stem SCW became bigger and was conducive to more water and mineral transport. Thus, there is reasonable to increase the productivity in *RLM1* knockout lines. RLM1 perhaps coordinates the balance of C and N, which is very similar to OsMYB61 (Gao et al., 2020). *RLM1* is highly expressed in the stems, and ectopic expression of *RLM1* affects both leaf morphology and plant height, indicating that *RLM1* perhaps participates in the gibberellin (GA) biosynthetic or signaling pathway. GA acts as the key hub between nitrogen and carbon (Gao et al., 2020), and *RLM1* may coordinate the nitrogen and carbon balance in plants.

## Data Availability Statement

The datasets presented in this study can be found in online repositories. The names of the repository/repositories and accession number(s) can be found at: https://www.ncbi.nlm.nih.gov/bioproject/PRJNA813008.

## Author Contributions

ZC and ST conceived the main study. ZZ and YC obtained the mutant and constructed the genetic population. PA and JW constructed the genetic transformation. XC, XS, and DL constructed the expression analysis and sub-cell localization. LZ constructed the yeast interaction. ZZ, XC, and ZC contributed to the data analysis. ZZ and TL designed and wrote the manuscript. All authors contributed to the article and approved the submitted version.

## Funding

This research was supported by the National Key Research and Development Program of China (2020YFA0907603 and 2020YFE0202300), NSFC (32001532), NSFC-CGIAR (31861143006), the Agricultural Science and Technology Innovation Program for TL, XC, and ZZ support, and the Fundamental Research Funds for Central Non-Profit of Institute of Crop Sciences for TL, XC, and ZZ support.

## Conflict of Interest

The authors declare that the research was conducted in the absence of any commercial or financial relationships that could be construed as a potential conflict of interest.

## Publisher’s Note

All claims expressed in this article are solely those of the authors and do not necessarily represent those of their affiliated organizations, or those of the publisher, the editors and the reviewers. Any product that may be evaluated in this article, or claim that may be made by its manufacturer, is not guaranteed or endorsed by the publisher.

## Footnotes

^1^ http://skl.scau.edu.cn

^2^ http://www.dna.affrc.go.jp/PLACE/

^3^ http://bioinformatics.psb.ugent.be/webtools/plantcare/html/

^4^ www.dna.affrc.go.jp/PLACE/?action=newplac

## Supplementary Material

Supplementary Table S1 Up regulated genes and down regulated genes in WT and *rlm1-D* lists.

Supplementary Table S2 List of RLM1 binding motifs.
