# Supplementary material for: RLM1, Encoding an R2R3 MYB Transcription Factor, Regulates the Development of Secondary Cell Wall in Rice
Source: Front Plant Sci. 2022 May 31;13:905111. doi: 10.3389/fpls.2022.905111 (PMC9194675; doi:10.3389/fpls.2022.905111)
Supplement: Supplementary file 4 [file Data_Sheet_1.docx]

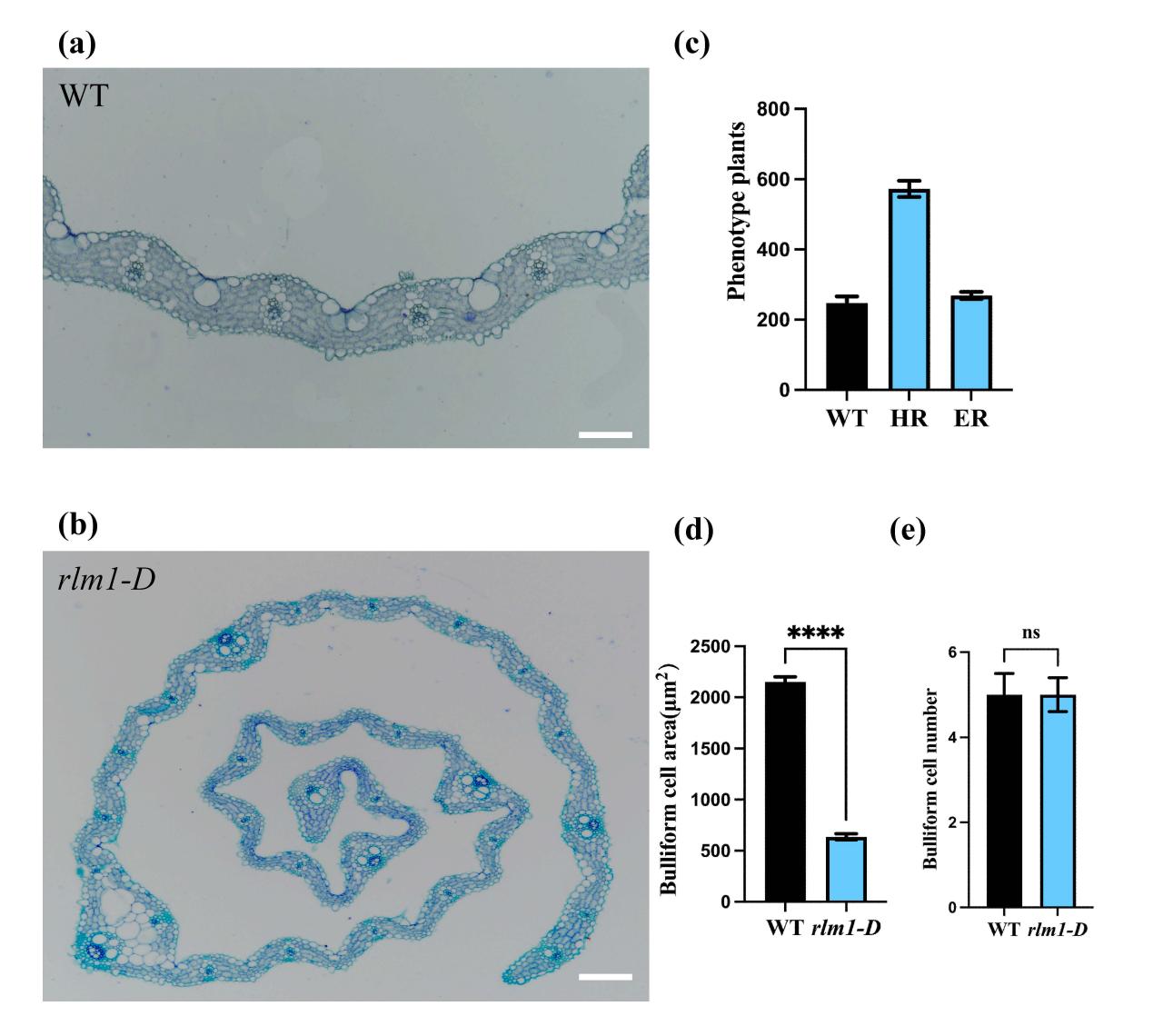


**Figure S1. Leaf blade comparison and** **cytological changes in WT plants and *rlm1-D* mutants.**

(a). Composite image of a mature leaf blade of WT plants. Bars = 20 µm.

(b). Composite image of a mature leaf blade of the *rlm1-D* mutant. Bars = 20 µm.

(c). Segregation ratio resulting from the crossing of heterozygous rlm1 plants: excessive rolling (ER)/half rolling (HF)/WT (WT) = 1/2/1.

(d-e). The *rlm1-D* showed a reduced size of bulliform cells, but the number of bulliform cells was normal in mature flag leaves compared with those of the WT.


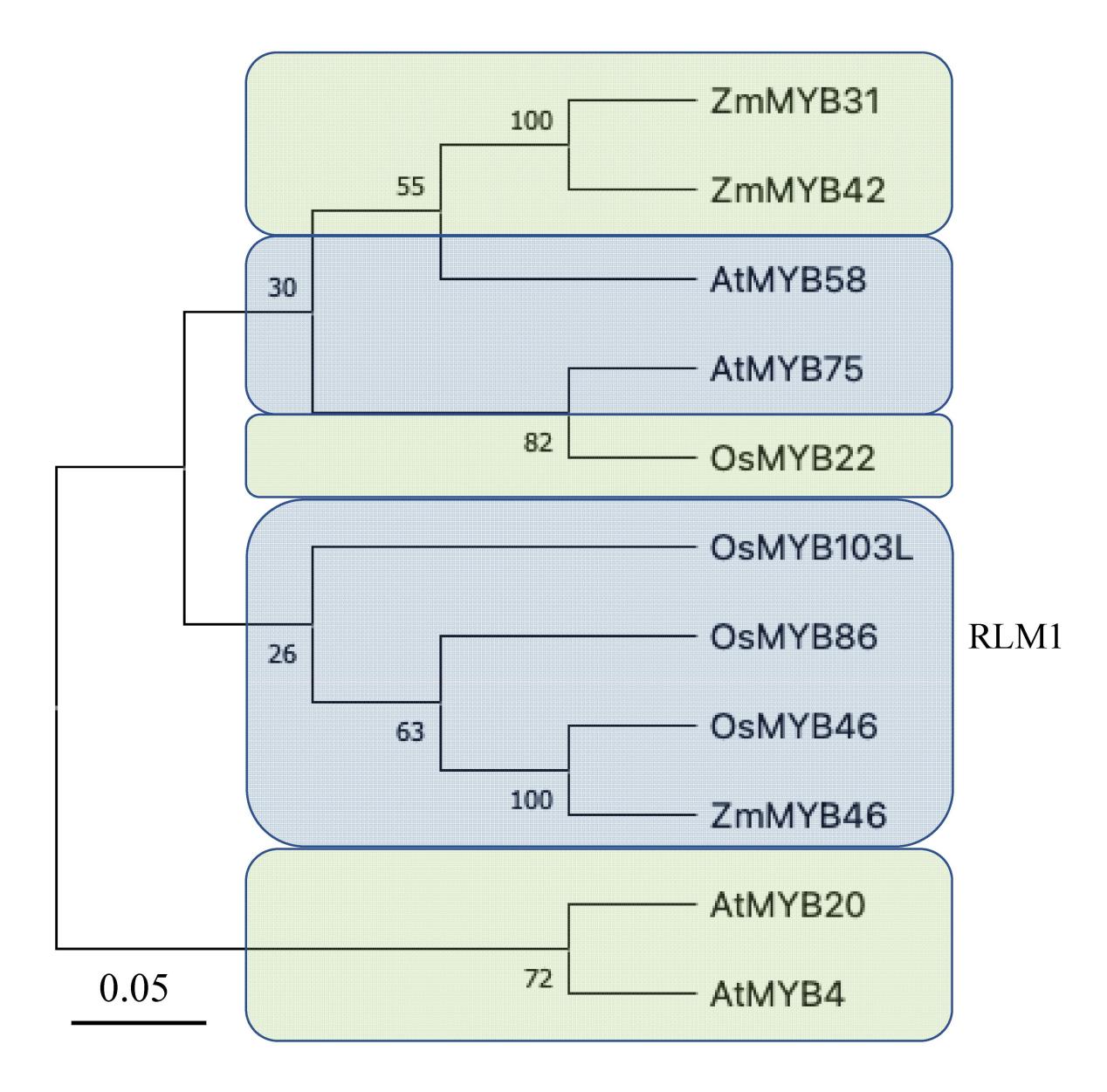


**Fig. S2. Phylogenetic tree showing the predicted relationships between OsMYB86 (RLM1) and homologous proteins in rice and Arabidopsis.** Full-length amino acid sequences of each protein were aligned using CLUSTALW and revised manually. The tree was constructed using the neighbor-joining method. Neighbor-joining phylogenetic tree analysis based on full-length protein sequences from the R2R3 MYB family members *Oryza sativa* and *Arabidopsis*.

**
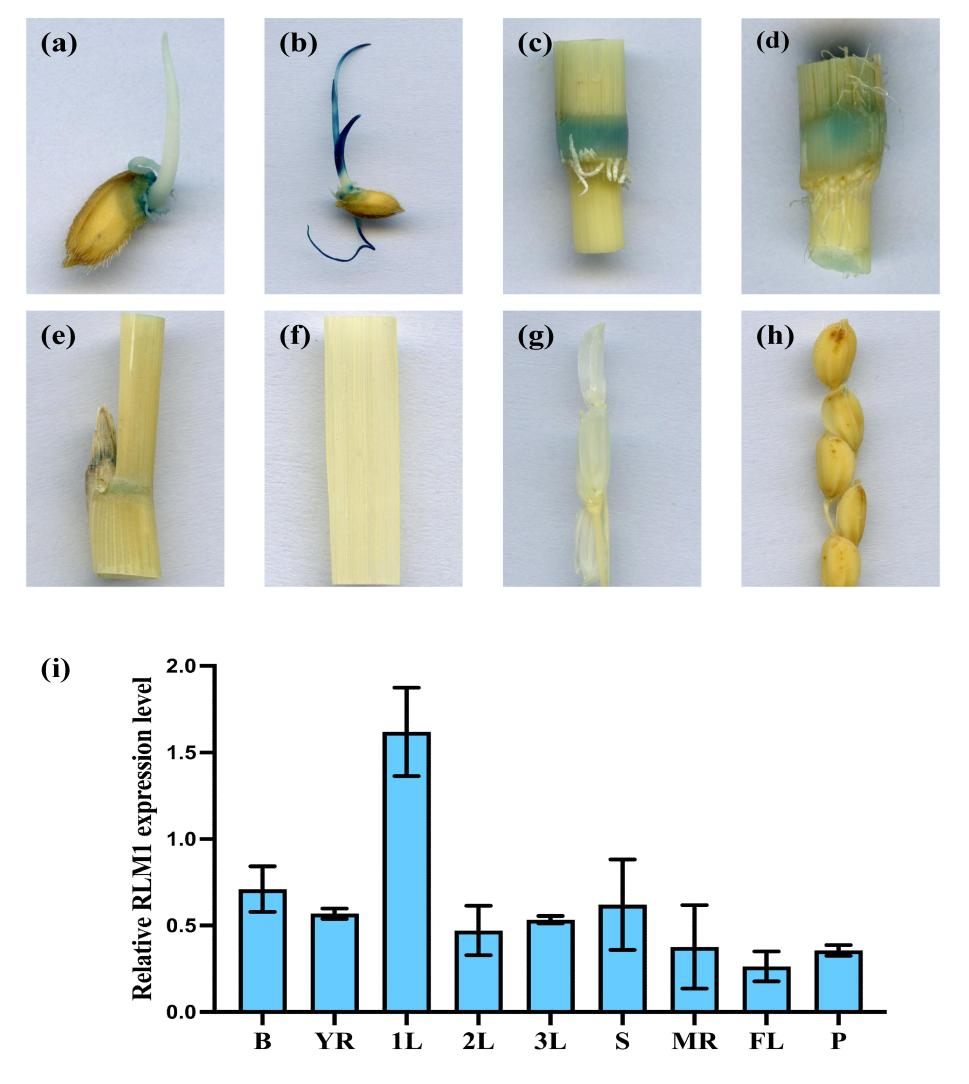
**

**Figure S3. Expression patterns of RLM1.**

(a-i). Transgenic plants containing the RLM1-pCAMBIA1391Z vector were stained with GUS dye.

(a): bud from germination after 3 days.

(b): bud from germination after 6 days.

(c): second internodes at the mature stage.

(d): fourth internodes at the mature stage.

(e): leaf ligule. (f): leaf blade; (g): young panicle.

(h): mature panicle. The results revealed deep staining of germinating buds and roots; at the mature stage, GUS staining was detected only in the internodes.

(i). Relative expression of RLM1 in the different tissues. B: bud; YR: young root; 1 L: first leaf; 2 L: second leaf; 3 L: third leaf; S: mature stem; MR: mature root; FL: flag leaf; P: panicle.


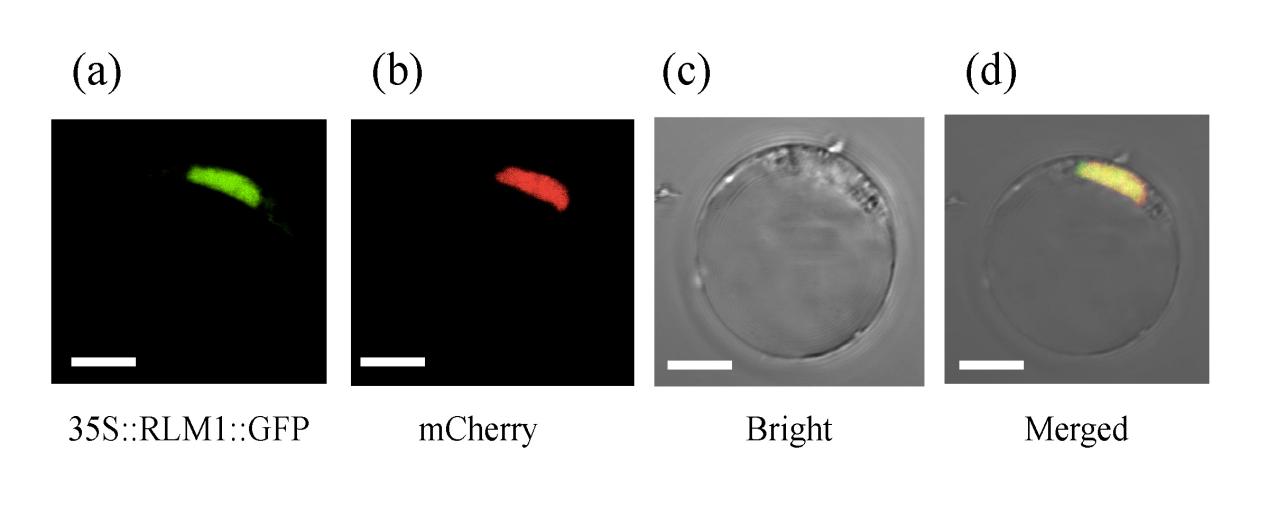


**Figure S4. Subcellular location of RLM1.**

(a-d) A 35S::RLM1:GFP vector was transformed into rice protoplasts, and the green fluorescent signal was visualized using confocal microscopy. OsMADS3, which localizes to the nucleus, was selected as a marker. The results revealed colocalization of the GFP signal with OsMADS3 fluorescence, indicating that RLM1 is mainly targeted to the rice nucleus. Bar = 100 µm.


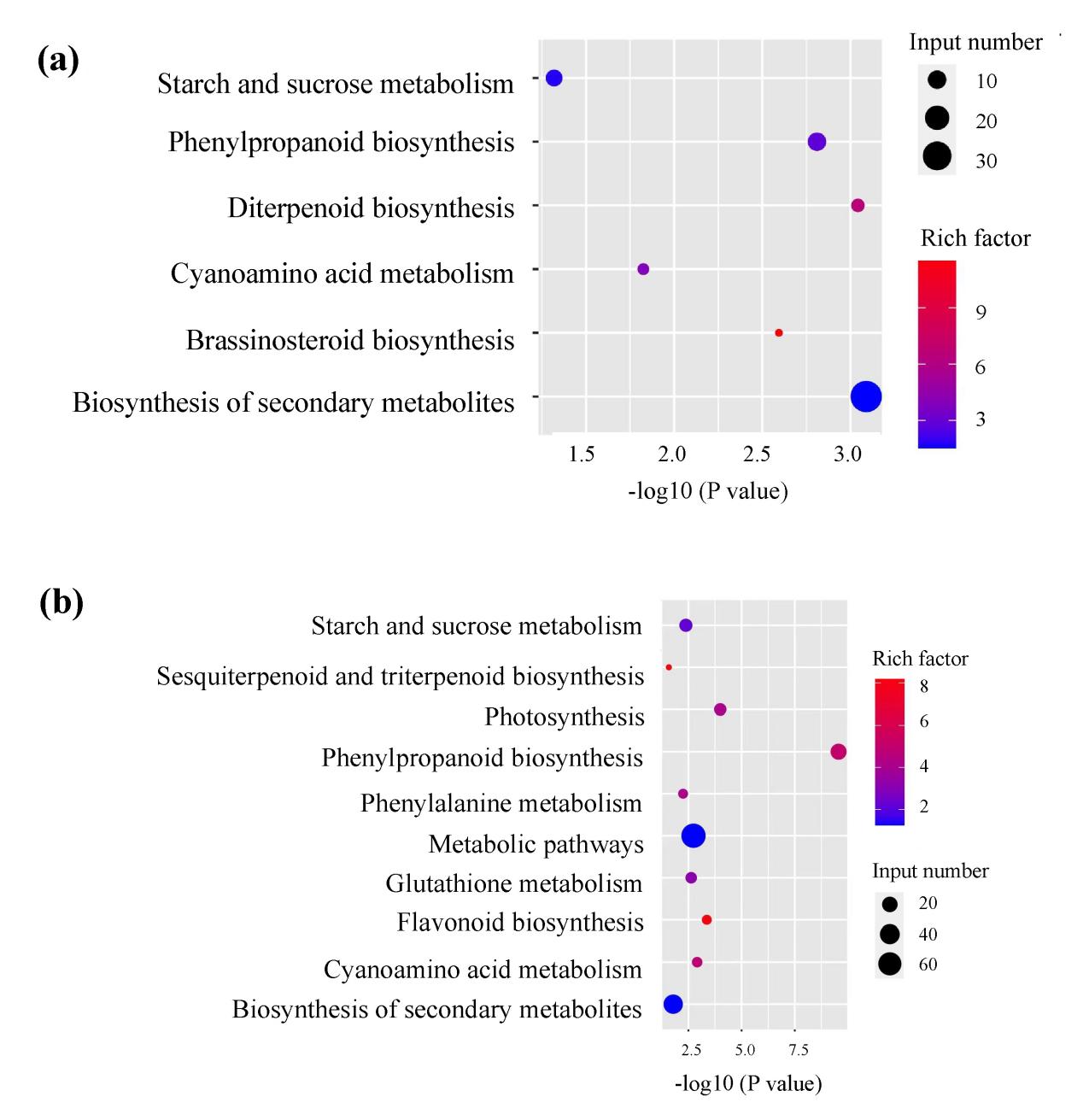


**Figure S5.** KEGG enrichment analysis of downregulated and upregulated genes differentially expressed between the *rlm1* -D and WT lines.

(a) KEGG enrichment analysis of downregulated genes differentially expressed between the *rlm1* -D and WT lines.

(b) KEGG enrichment analysis of upregulated genes differentially expressed between of the *rlm1* -D and WT lines. The X-axis indicates the enrichment ratio (the ratio of the number of genes annotated to an entry in the selected gene set to the total number of genes annotated to the entry of the species). The Y-axis indicates the KEGG pathway, and the size of the bubble indicates the number of genes. The colour represents the enriched Q-value; the darker the colour is, the smaller the Q-value is.


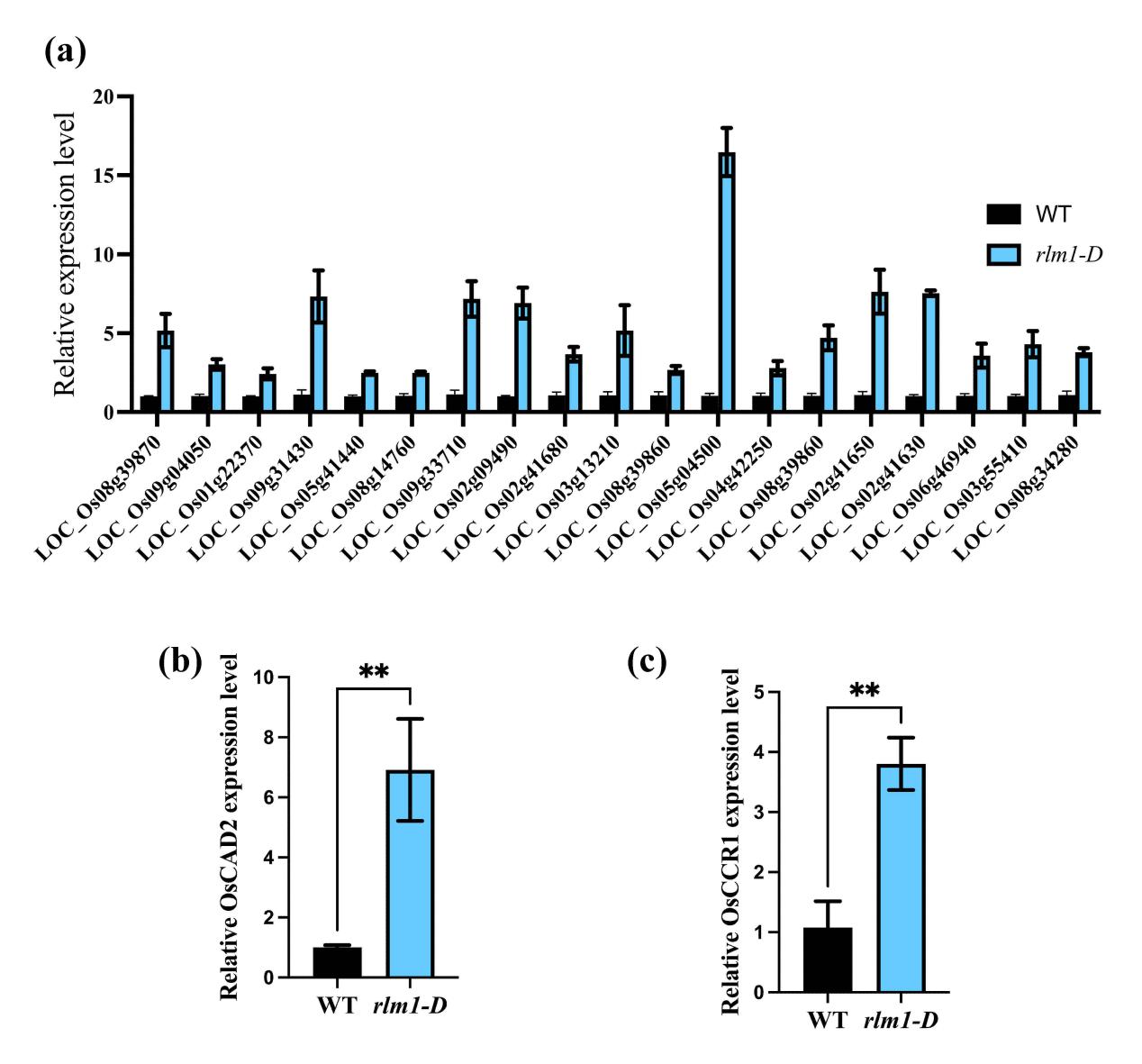


**Figure S6.** The expression levels the phenylpropanoid metabolic pathway-related genes in WT and *rlm1* -D.

(a) The expression levels of phenylpropanoid metabolic pathway-related genes in WT and *rlm1* -D plants at the late-tillering stage were measured by qRT-PCR (n=3).

(b) Expression analysis of OsCAD2 in WT and *rlm1* -D.

(c) Expression analysis of OsCCR1 in WT and *rlm1* -D.


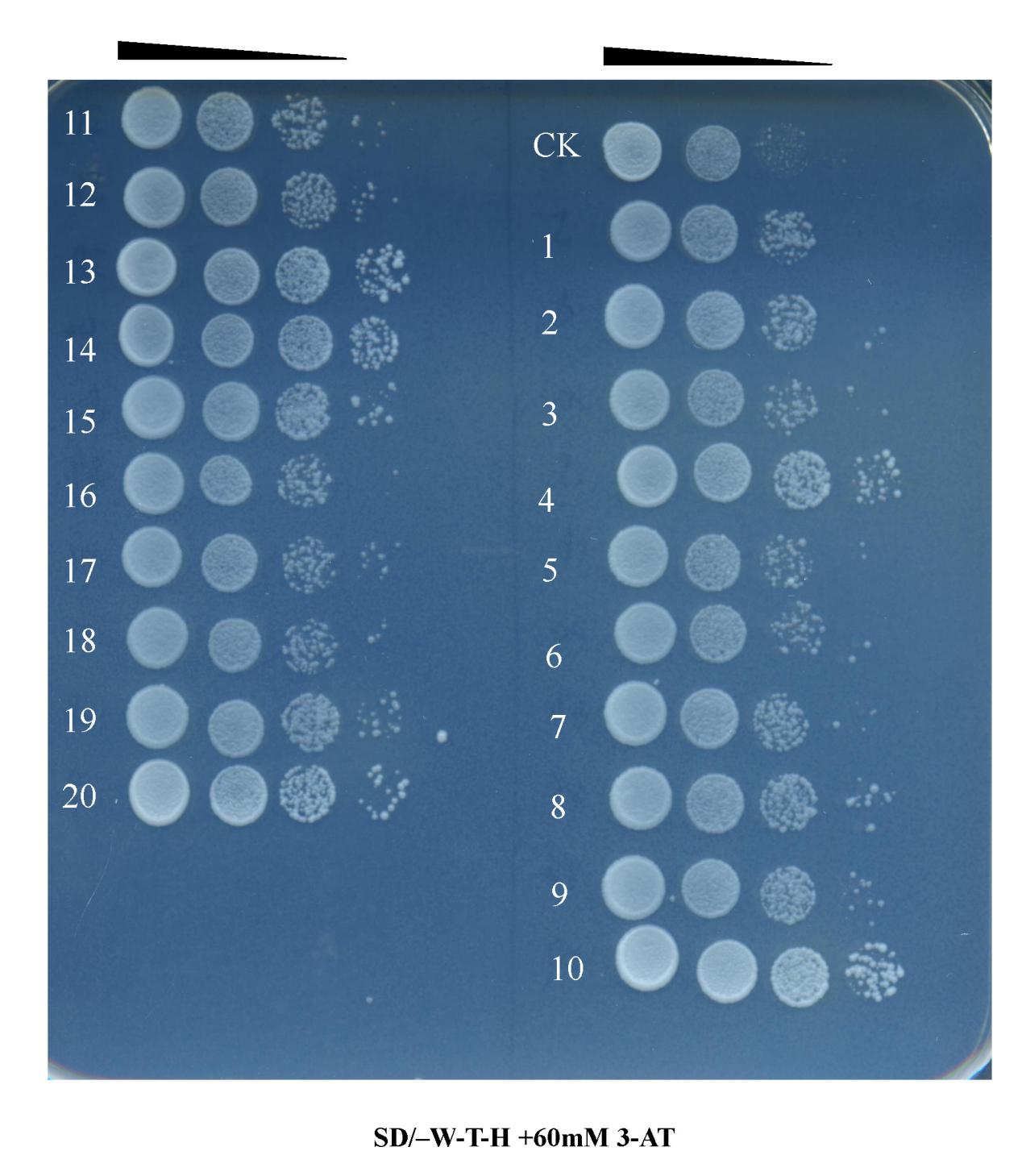


**Figure S7. Determination of the core sequence of the novel motif recognized by RLM1.**

The sequences (1-20) were allowed to interact with RLM1 via Y1H assays to determine the core motif. The triangles indicate the dilutions (1/1, 1/10, 1/100, 1/1000) of the yeast. The core sequences are shown in Table S2. CK represents p53-AD and p53-His2.

**
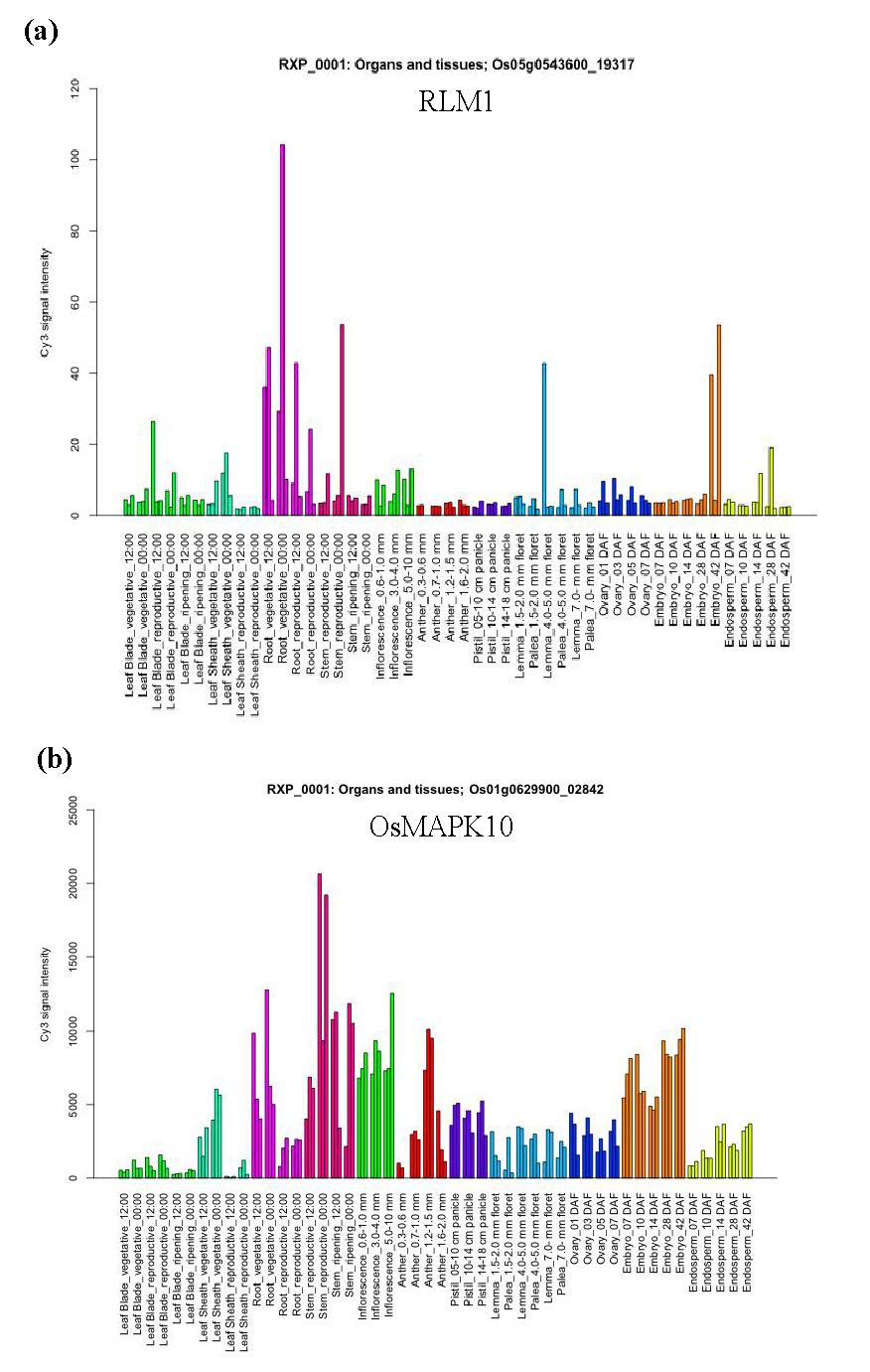
**

**Figure S8**. The expression patterns of RLM1 (up) and OsMAPK10 (down) in the RiceXPro database (https://ricexpro.dna.affrc.go.jp/). RLM1 and OsMAPK10 exhibited the highest expression in the roots and stems.





**Figure S9. CRISPR/Cas9-induced mutations in *OsMAPK10***

(a) Schematic map of the genomic region of *OsMAPK10* and the sgRNA target site. The arrow shows the sgRNA target site on the *OsMAPK10* genomic sequence, and the PAM motif (NGG) is shown in red. The blue boxes represent *OsMAPK10* exons, and the black lines indicate intron sequences.

(b) Amino acid alignment around the sgRNA target region showing the predicted peptide sequence of the WT and mutant alleles. The site of the predicted frame-shifted sequence is underlined, and new stop codons are shown by asterisks.

(c) Phenotypes of WT plants and OsMAPK10*-m1* and *OsMAPK10-m2* homozygous mutants at the tillering stage. (bar = 5 cm)

(d) Analysis of *OsMAPK10* expression levels in WT plants and OsMAPK10*-m1* and *OsMAPK10-m2* homozygous mutants.

(c). Lignin content measurements in the second internodes from three-month-old WT plants and OsMAPK10*-m1* and *OsMAPK10-m2* homozygous mutants.

(d). Cellulose content measurements in the second internodes from three-month-old WT plants and OsMAPK10*-m1* and *OsMAPK10-m2* homozygous mutants.

(e). Lignin content measurements in leaf tissue of WT plants and OsMAPK10*-m1* and *OsMAPK10-m2* homozygous mutants.

(f). Cellulose content measurements in leaf tissue of WT plants and OsMAPK10*-m1* and *OsMAPK10-m2* homozygous mutants.


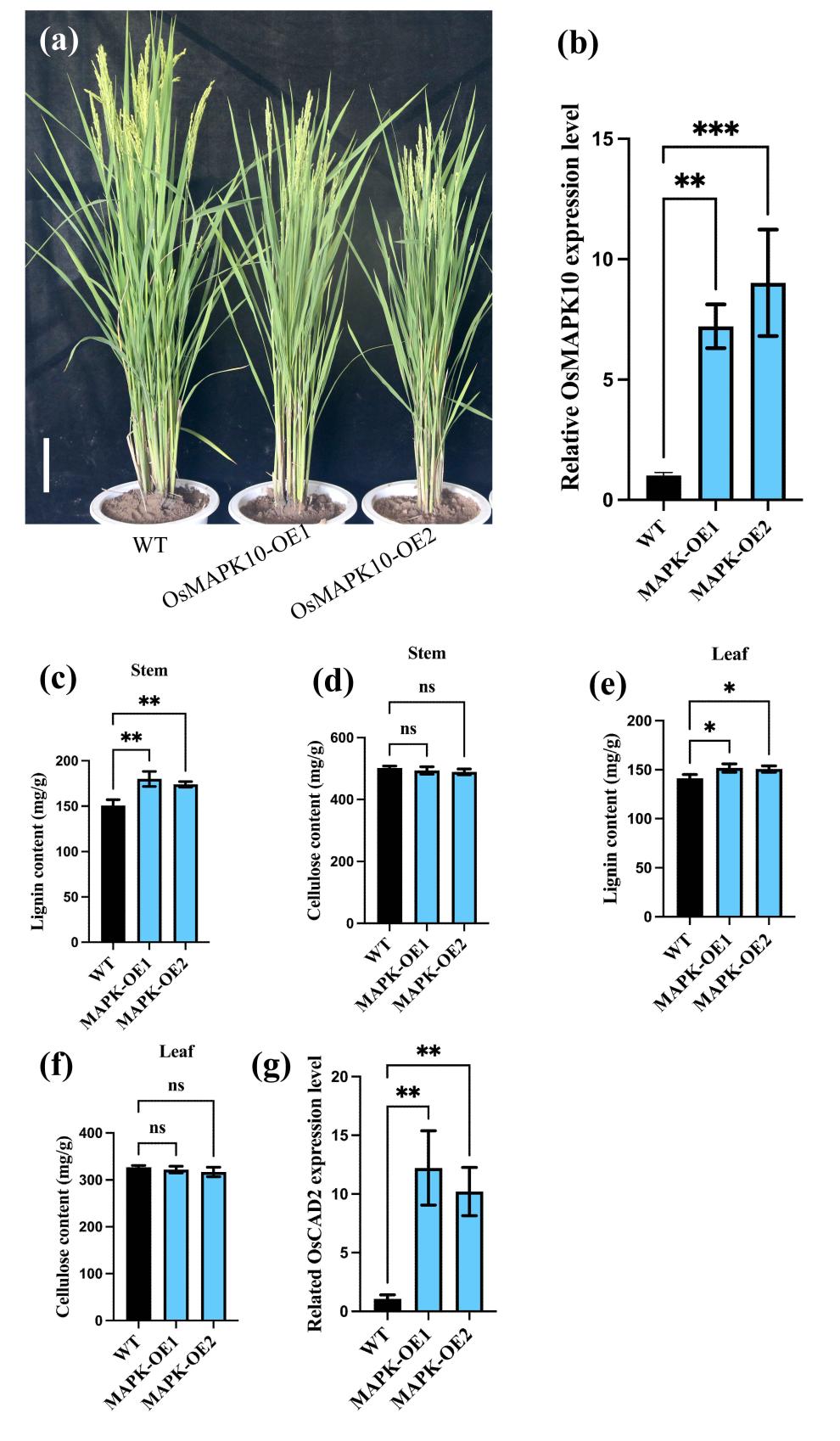


**Figure S10. Analysis of OsMAPK10 overexpression in the WT background.**

(a). Comparison of WT and OsMAPK10 overexpression plants (OE1, OE2) at the heading stage. (bar = 5 cm)

(b). Analysis of OsMAPK10 expression levels in WT and OsMAPK10 overexpression plants (OE1, OE2)

(c). Lignin content measurements in the second internodes from three-month-old WT and OsMAPK10 overexpression plants (OE1, OE2).

(d). Cellulose content measurements in the second internodes from the three-month-old WT and OsMAPK10 overexpression plants (OE1, OE2).

(e). Lignin content measurements in WT, OE1, and OE2 leaf tissue.

(f). Cellulose content measurements in WT, OE1, and OE2 leaf tissue.

(g). Analysis of OsCAD2 expression levels in WT and OsMAPK10 overexpression plants (OE1, OE2).


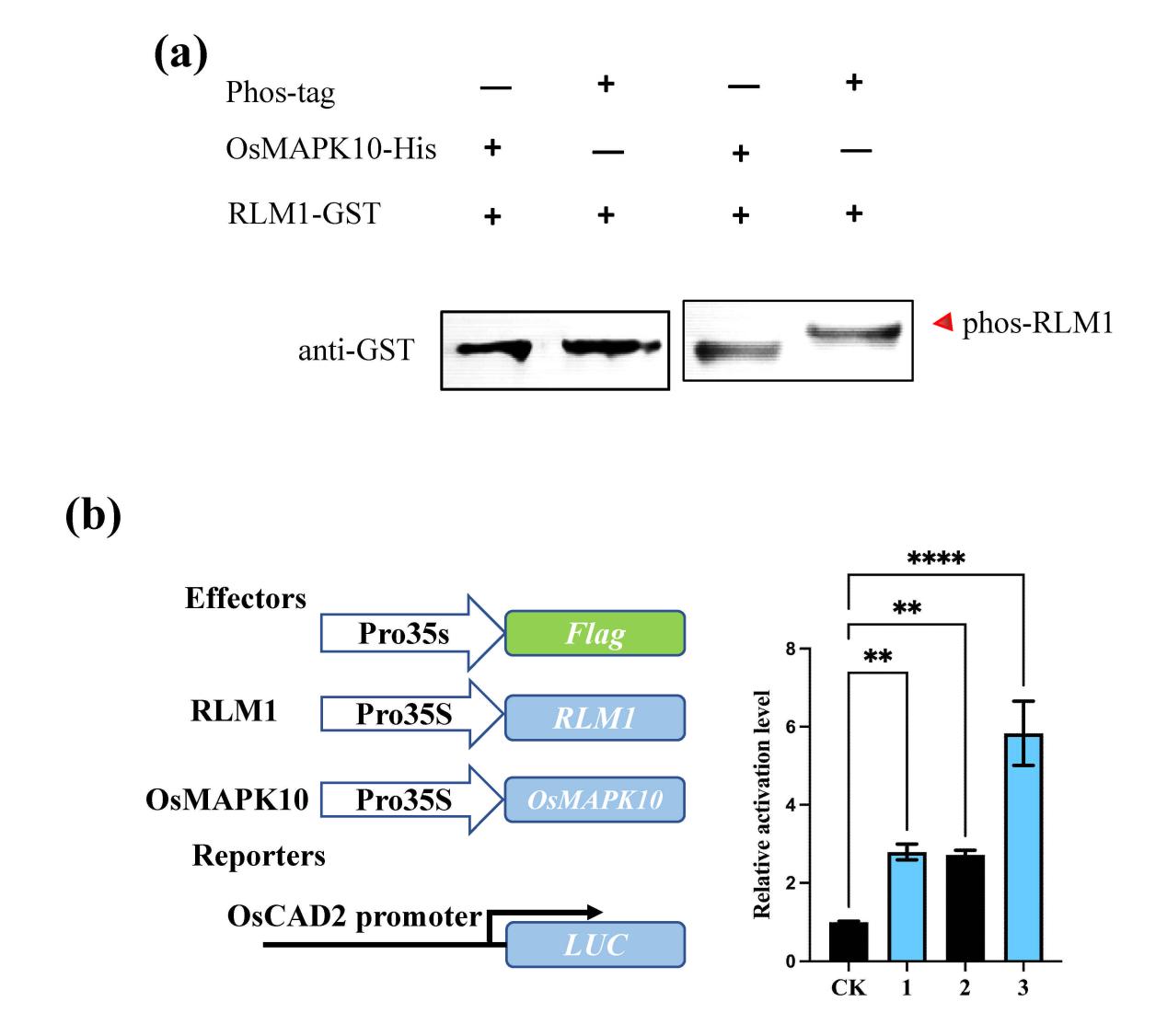


**Figure S11. OsMAPK10 phosphorylates RLM1 and enhances RLM1 activation activity.**

(a). OsMAPK10 phosphorylates RLM1 proteins. Western blotting was conducted using anti-GST antibodies. The red arrow indicates the phosphorylation band of RLM1 when Phos-Tag was added.

(b). OsMAPK10 helped RLM1 to activate OsCAD2.


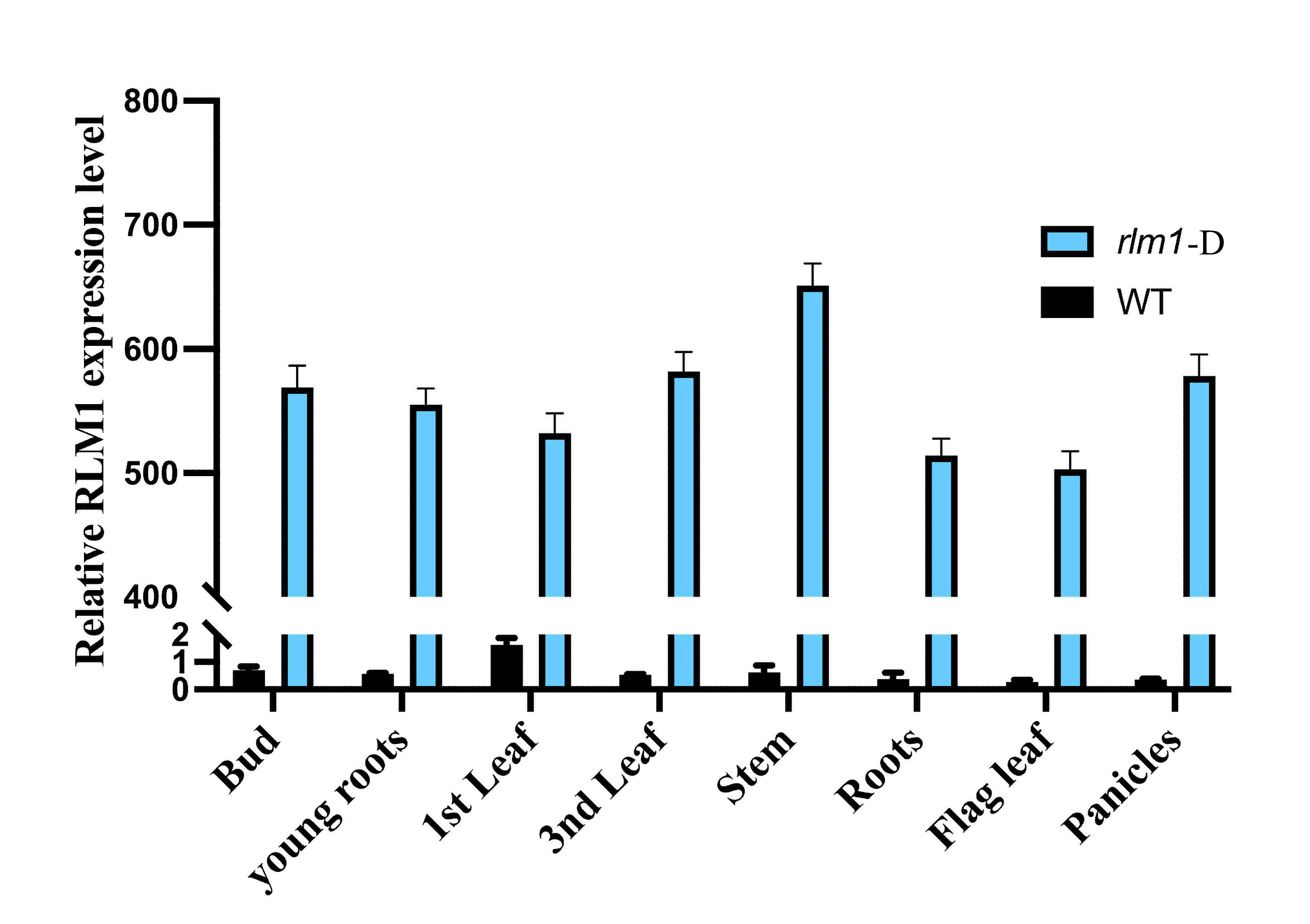


**Figure S12**. Relative expression of RLM1 in the different tissues in WT and *rlm1-D*. Tissues included bud; YR: young root; 1st: first leaf; 3nd: third leaf; S: mature stem; Roots: mature root; Flag leaf; panicle.
